# Human pluripotent stem cell fate trajectories toward lung and hepatocyte progenitors

**DOI:** 10.1016/j.isci.2023.108205

**Published:** 2023-10-14

**Authors:** Chaido Ori, Meshal Ansari, Ilias Angelidis, Ruth Olmer, Ulrich Martin, Fabian J. Theis, Herbert B. Schiller, Micha Drukker

**Affiliations:** 1 Institute of Stem Cell Research, Helmholtz Munich, Neuherberg, Munich, Germany; 2 Comprehensive Pneumology Center Munich (CPC-M), Institute of Lung Health and Immunity (LHI), Helmholtz Munich, Member of the German Center for Lung Research (DZL), Munich, Germany; 3 Department of Computational Health, Institute of Computational Biology, Helmholtz Munich, Munich, Germany; 4 Leibniz Research Laboratories for Biotechnology and Artificial Organs (LEBAO), Department of Cardiothoracic, Transplantation and Vascular Surgery (HTTG), Hannover Medical School, 30625 Hannover, Germany; 5 Biomedical Research in Endstage and Obstructive Lung Disease (BREATH), Member of the German Center for Lung Research (DZL), Hannover Medical School, 30625 Hannover, Germany; 6 REBIRTH-Research Center for Translational and Regenerative Medicine, Hannover Medical School, 30625 Hannover, Germany; 7 TUM School of Life Sciences, Technical University of Munich, Munich, Germany; 8 Institute of Experimental Pneumology, LMU University Hospital, Ludwig-Maximilians University, Munich, Germany; 9 Division of Drug Discovery and Safety, Leiden Academic Centre for Drug Research (LACDR), Leiden University, Leiden, the Netherlands

**Keywords:** Molecular biology, Cell biology, Developmental biology

## Abstract

In this study, we interrogate molecular mechanisms underlying the specification of lung progenitors from human pluripotent stem cells (hPSCs). We employ single-cell RNA-sequencing with high temporal precision, alongside an optimized differentiation protocol, to elucidate the transcriptional hierarchy of lung specification to chart the associated single-cell trajectories. Our findings indicate that Sonic hedgehog, TGF-β, and Notch activation are essential within an ISL1/NKX2-1 trajectory, leading to the emergence of lung progenitors during the foregut endoderm phase. Additionally, the induction of HHEX delineates an alternate trajectory at the early definitive endoderm stage, preceding the lung pathway and giving rise to a significant hepatoblast population. Intriguingly, neither KDR+ nor mesendoderm progenitors manifest as intermediate stages in the lung and hepatic lineage development. Our multistep model offers insights into lung organogenesis and provides a foundation for in-depth study of early human lung development and modeling using hPSCs.

## Highlights

• Optimized differentiation protocol for derivation of lung progenitors
• Gene expression dynamics from pluripotency to the NKX2-1 lung progenitor state
• Identification of transcriptional regulators of lung and hepatoblast specification
• Notch and TGF-β crosstalk during specification of the foregut to lung progenitors

## Graphical abstract

**Figure:**
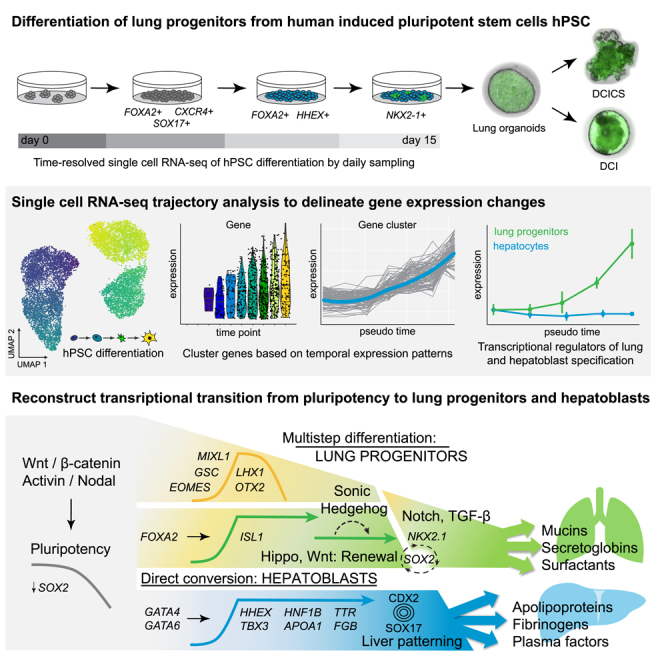

## Introduction

A primary goal of stem cell biology is to understand how the intricate regulation of cellular differentiation by cascades of gene regulatory networks and signaling pathways gives rise to the structure and function of organs. This knowledge is fundamental to human pluripotent stem cell (hPSC)-based approaches that model human development and congenital pathologies *in vitro*, and to devise regenerative therapies. The elucidation of molecular mechanisms governing foregut endoderm (FE) formation is of particular interest for biomedical research, as the FE gives rise to several organs, including the lung and the liver, that are relevant for disease modeling, drug screening assays, and regenerative applications. Various definitive endoderm (DE) progenitors, and the genes and pathways that regulate their specification, need to be better characterized in order to effectively control the differentiation of cells that represent endodermal tissues *in vitro,* such as epithelial cells of the airways and hepatocytes.

Single cell genomics is the method of choice for analyzing cell-cell heterogeneity and cellular differentiation trajectories in development.^1^^,^^2^^,^^3^^,^^4^ Single cell RNA sequencing (scRNA-seq) when applied to differentiation protocols of hPSCs can thus used to understand aspects of human development from *in vitro* studies. For instance, the molecular “roadmap” governing the development of functional human islet beta cells has been characterized by applying scRNA-seq time-series experiments.^5^ While embryological studies in mice have been instrumental in revealing the major stages and genes involved in FE development, insight into the early steps of human lung specification is largely pending. Recent studies have taken important steps in this direction by characterizing signaling pathways that regulate the differentiation of hPSC-derived NKX2-1+ progenitors into alveolar epithelial type 2 (AT2) cells.^6^ NKX2-1 is a transcription factor (TF) that is essential for lung and thyroid development in the foregut,^7^ and hence, expression of this gene serves as a hallmark to characterize hPSC-derived progenitors that can give rise to functional lung cells.^8^^,^^9^^,^^10^ Nevertheless, the intermediate states and mechanisms prompting the commitment of NKX2-1+ lung progenitors during endoderm differentiation have not been characterized in detail. In this regard, it is not well understood which mechanisms lie upstream of NKX2-1, and why mixtures of cells that express NKX2-1 and hepatic markers such as FGB are being produced by the current state of the art differentiation protocols.^10^

Current protocols for lung progenitor differentiation from hPSCs utilize a stepwise application of signaling cues that are known to be important in lung development. Treatment of hPSCs with Activin/Nodal in conjunction with activation of Wnt/β-catenin signaling promotes the differentiation of DE, followed by transient inhibition of endogenous Activin/Nodal and BMP signaling by “dual SMAD inhibition”, which promotes further commitment to the FE.^11^ In the last step Wnt/β-catenin is reactivated in conjunction with RA, BMP4, and FGF10 treatment, resulting in the formation of NKX2-1+ progenitors.^11^^,^^12^ NKX2-1+ progenitors have been shown to mimic early lung organogenesis as they can produce alveolar and bronchiolar spheroids that express proteins involved in respiratory functions including surfactants.^13^^,^^14^^,^^15^^,^^16^

Differentiation protocols of hepatic lineages and lung progenitors are similar in the initial activation of the Activin/Nodal pathway to specify the DE. However, contrary to lung differentiation protocols, hepatic induction involves treatment with FGF2 or FGF2+BMP4 in the DE phase.^17^ In the second stage, some protocols utilize SMAD inhibition similarly to lung differentiation protocols,^11^ while others utilize HGF.^18^ The disparities in the early induction of DE raise questions about the origin and identity of the hepatic cells that are neighboring the NKX2-1+ progenitors in lung differentiation protocols. Moreover, in this regard it is not resolved whether hepatic KDR+ progenitors^19^ or mesendoderm precursors^20^ are transient states in lung progenitor differentiation protocols.

The stalk and epithelial tips of human fetal lungs have recently been characterized by RNA sequencing,^21^ and a cell atlas for the adult human lung has been created by scRNA-seq.^22^^,^^23^ How NKX2-1+ progenitors are being formed during development remains an open question. Using time-series scRNA-seq, we model the hierarchy of gene expression changes along a trajectory from hPSCs to NKX2-1+ lung progenitors. Our model illustrates how timed inputs of Activin A/Nodal, Wnt/β-catenin, Hedgehog, and TGF-β pathways promote lung progenitor differentiation. We have discovered a novel early role for Notch signaling in this process. Surprisingly, we also found that the emergence of hepatoblasts can take place directly from early DE, prior to the formation of FE, when Activin/Nodal and Wnt/β-catenin signaling are at their peak.^24^ We have characterized molecular details of this branching event that specifies lung progenitors versus hepatoblast identity. The transcriptional roadmap established here thereby provides an important resource for understanding the origin and abnormal development of the human respiratory system and hepatic lineages.

## Results

### Parallel differentiaiton of human PSCs to lung progenitors and early liver cells

**Figure 1:**
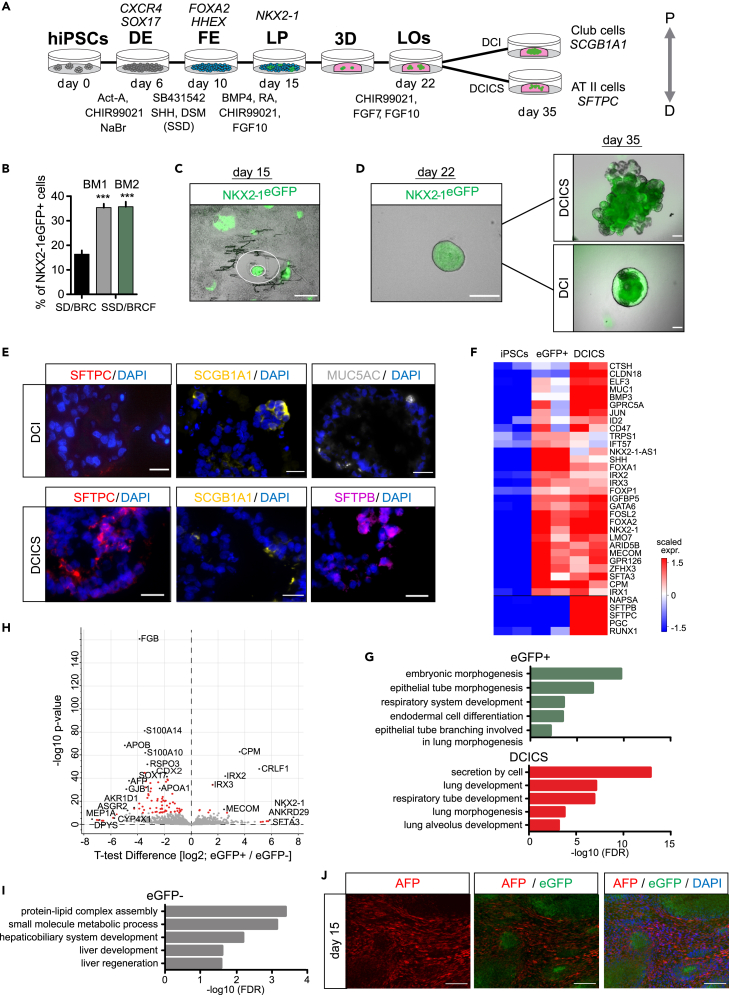
Differentiation of lung progenitors from hPSCs (A) A schematic illustration of the differentiation protocol used in this study to produce eGFP+ lung progenitor cells with spheroids and organoids the on right. (B) Quantification of the number of eGFP+ cells by flow cytometry on day 15 of differentiation; conditions with and w/o combined FGF10+SHH treatment in defined basal media DMEM/F12 and IMDM (FE-BM1 and BM2, respectively). SD stands for SB431542 and Dorsomorphin, while SSD stands for SD plus SHH for FE. Accordingly for LP stage induction, BRC stands for BMP4, RA and CHIR99021 while BRCF for BRC plus FGF10. Bars represent the mean +SD, n = 3 biological replicates, ∗∗∗p < 0.001 conducted using an unpaired one-tailed t-test. (C and D) Fluorescence microscopy images showing day 15 NKX2-1eGFP+ cell sectors (C), and a representative day 22 spheroid produced by embedding a colony in Matrigel, along with the further growth of spheroids (Scale bar: 200 μm) observed on day 35 with treatments as indicated (D). (E) Immunofluorescence staining of day 35 DCI and DCICS spheroid-organoids for SFTPC, SFTPB, SCGB1A1, and MUC5AC (scale bar: 20μm). (F) A heatmap displaying the expression of developmental markers and genes important for the function of the lung based on the bulk RNA-Seq analysis of the indicated conditions (p value <0.05, scale displays normalized log2 expression). (G) Gene Ontology (GO) term enrichment analysis of differentially expressed genes in eGFP+ and DCICS spheroid-organoids relative to undifferentiated *NKX2-1*^*eGFP/+*^ cells (p value <0.05, GO term FDR <0.05). (H and I) A volcano plot showing differentially expressed genes comparing the eGFP+ and eGFP- sorted populations on day 15 (H), and the corresponding GO terms of the eGFP- population (I). (J) Immunostaining for AFP (liver marker), imaging of eGFP+ cells (lung progenitors), and DAPI staining on day 15 of differentiation (scale bars: 100 μm). DE - definitive endoderm; FE - foregut endoderm; LP - lung progenitors; LOs - lung organoids.

In the mouse embryo, the formation of the primitive streak (PS) marks the stage where pluripotent cells commit to becoming precursors of the germ layers including the DE. The differentiation of DE in this process relies on the induction of TFs, including *Sox17*, *Foxa2*, and *Eomes*, as well as primitive streak TFs including *Mixl1*, *Goosecoid* (*Gsc*), and the down-regulation of pluripotency genes.^25^^,^^26^^,^^27^^,^^28^ The TFs that subsequently promote the anteriorization of the primitive gut tube leading to the induction of *NKX2-1*, are less characterized both in the mouse model and the *in vitro* differentiation of hPSCs. To investigate mechanisms underlying the formation of lung foregut precursors in human development, we optimized a differentiation protocol that utilizes stepwise activation and inhibition of Activin/Nodal and Wnt/β-catenin signaling,^10^^,^^13^^,^^16^ along with supplementation of sonic hedgehog (SHH) and FGF10 at the second stage, as these cues are essential in foregut and lung bud development (Figure 1A).^29^^,^^30^^,^^31^

To monitor the appearance of lung progenitors we used a human induced PSC (hiPSC) line integrated with the *eGFP* gene downstream of the endogenous promoter of *NKX2-1* (*NKX2-1*^*eGFP/+*^).^32^ Our protocol assessment showed that SOX17, FOXA2, and cell surface markers that are associated with DE, namely, CXCR4, CKIT, EPCAM were markedly up-regulated at day 6 of the differentiation protocol (Figures S1A and S1B). The formation of eGFP+ progenitors became apparent between days 13–15, and their number significantly increased by supplementation of SHH and FGF10, which also improved the overall cell survival (Figure S1C). We compared two types of basal media, namely, DMEM/F12 and IMDM-based (BM1 and BM2 as described in the experimental procedures), showing 100% success rate with IMDM and an average efficiency of approximately 35% NKX2-1+ cells located in regions of eGFP expression (Figures 1B, 1C, S1C, and S1D). Since *NKX2-1* is expressed in the fetal lung, as well as in the thyroid and forebrain progenitors,^7^ we analyzed the expression of *PAX8* and *PAX6* which are representative markers of the latter tissues. The low expression levels of these markers on day 15 indicated that the *NKX2-1* signal represented the formation of lung progenitors but not the other tissues (Figure S1E).

Next, we analyzed the developmental potential of putative eGFP+ lung progenitors using spheroid 3D culture assays. We embedded clusters of eGFP+ cells from day 15 of differentiation in Matrigel and supplemented the media with CHIR99021 (CHIR), FGF7, and FGF10 (Figure 1A), to promote the proliferation of lung progenitors in suspension culture.^10^ This approach led to the outgrowth of spherical structures, which tripled in size within 7 days and maintained the expression of eGFP (Figures 1D, S1F, and S1G). Subsequent treatment with dexamethasone, cAMP and IBMX (DCI), known to promote the maturation of the fetal lung,^33^^,^^34^ induced the expression of genes and proteins associated with club cells and goblet cells in the proximal region of the lung, namely, SCGB1A1 and MUC5AC (Figures 1E and S1F–S1H). When Wnt/β-catenin pathway was activated by CHIR, the spheroids developed branches (DCIC), and further, when combined with inhibition of TGF-β by SB431542 (DCICS), the spheroids grew substantially larger to an average size of 1.6 mm by day 35. They also exhibited branches and markers of alveolar epithelial type II cells, namely, SFTPC and SFTPB, and diminished expression of *SCGB1A1* and *MUC5AC* compared to DCI (Figures 1E and S1F–1H).

To characterize the developmental stage, the differentiation potential of NKX2-1+ progenitors, and the basis of the heterogeneity in eGFP expression, we sequenced the mRNAs of undifferentiated cells, sorted eGFP+ and eGFP- populations and DCICS organoids (Figures S1I and S1J). Genes that have been implicated in the formation of respiratory epithelial cells in the lung, namely, *FOXA2*, *FOXA1*, *FOXP1*, and *NKX2-1*,^35^^,^^36^ were highly expressed in the eGFP+ cells and DCICS organoids (Figure 1F). GO term analysis furthermore revealed enrichment of genes involved in embryonic respiratory lung morphogenesis and alveolar development in eGFP+ progenitors and DCICS organoids (Figure 1G). The organoids additionally exhibited markers of early and late branching morphogenesis and differentiation of the distal lung, including *RUNX1, MUC1*, *SFTPC*, *SFTPB*, *CLDN18*, and *NAPSA* (Figure 1F), and their expression pattern closely resembled that of published transcriptomes of the alveolar tips of human fetal lungs, but was less similar to the stalk of fetal lungs (Figure S1K).

Analysis of eGFP- cells revealed that the eGFP-NKX2-1- population expressed fetal liver genes, including apolipoproteins (e.g., *APOA1* and *APOB*) fibrinogen (*FGB*), the plasma protein alpha fetoprotein (*AFP*),^17^^,^^18^ and GO term analysis revealed the enrichment of fetal liver development, regeneration and metabolism categories (Figures 1H and 1I). To confirm the coexistence of lung progenitors and hepatocytes on day 15 of the differentiation protocol, cultures were immunostained by AFP and eGFP antibodies. This showed mutually exclusive expression of eGFP and AFP, and the presence of lung progenitors and hepatoblasts in separate clusters (Figure 1J). Taken together, we concluded that co-induction of lung and liver fates took place during the differentiation of lung progenitors from hPSCs, raising the question of the mechanisms that drive the mutually exclusive specification of these lineages from FE.

### Time-resolved single cell RNA-seq of hPSC differentiation

**Figure 2:**
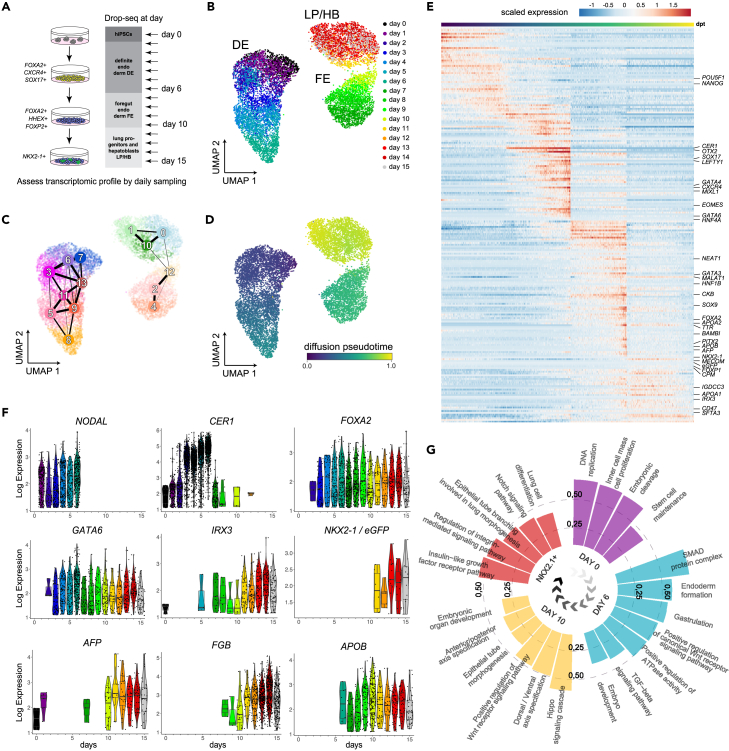
Time-resolved analysis of hPSC differentiation using scRNA-seq shows distinct hierarchy of gene expression changes (A) A schematic illustration of daily sampling for scRNA-seq during 15 days of the lung progenitor differentiation. (B) The time of sampling is color coded on the UMAP projection of scRNA-seq transcriptomes (HB: hepatoblasts). (C) The connectivity of distinct Louvain clusters as determined by graph abstraction (PAGA) is shown on the UMAP projection. (D) The UMAP is colored by the stage-wise inferred pseudotime; darker regions (toward blue) indicate starting points and brighter colored regions (toward yellow) indicate ending points. (E) A heatmap illustrates peaks of gene expression ordered by the pseudotime of differentiation. Selected developmental markers of the lung and the liver are displayed. (F) Violin plots of stage specific marker genes are shown for the real time points of sampling in days. (G) Circular bar chart showing enriched gene categories taken from UniPort Keywords, GO terms and KEGG pathways corresponding to the pluripotent cells, DE, FE and NKX2-1+ progenitors (FDR <5%).

To chart a comprehensive map of the transcriptional states underlying the differentiation of lung progenitors and hepatoblasts (HB) in parallel, we performed a 16-day time-series scRNA-seq using the Drop-Seq workflow.^37^ Single cell suspensions were processed daily, resulting in the analysis of a total of 10,667 cells that passed quality standards and were used for downstream analysis (Figures 2A and S2A; Tables S2 and S3). First, we used the Uniform Manifold Approximation and Projection (UMAP) and Partition-based Graph Abstraction (PAGA) analysis^38^ to project the gene expression data onto two dimensions and to perform an assessment of the connectivity of the obtained cell clusters (Figures 2B, 2C, and S2A–2C). This revealed three major domains in the high dimensional gene expression manifold corresponding to the three stages of the differentiation protocol (Figures 2B, 2C, and S2A).

To model the dynamics of gene expression along the entire trajectory we used pseudotime inference (Figure 2D), which was in a good agreement with the time points of sampling (Figure S2D). Unsupervised hierarchical clustering of the genes along the pseudotime ordering revealed stage specific markers, including TFs expressed in undifferentiated PSCs e.g., *POU5F1* (*OCT4*) and *NANOG*; DE factors including e.g., *SOX17*, *MIXL1*, *EOMES*, *CER1*, *CXCR4*, and *LEFTY1*; TFs characteristic for the FE e.g., *FOXA2*, and *FOXP1*;^36^ and genes important for the formation of lung progenitors e.g., *NKX2-1* and *IRX3* emerging in the latest stage (Figures 2E, S2B, S2E, and S2F; Table S5).^7^^,^^39^ The pseudotime was consistent with the consecutive expression of DE, FE, and lung progenitor markers, e.g., *NODAL*, *CER1*, *FOXA2*, *IRX3*, and *NKX2-1* during the time course (Figure 2F). Importantly, genes that are characteristic of hepatoblasts and hepatocytes, including *GATA6*, *AFP*, *FGB*, and *APOB* were markedly up-regulated, some already during the second stage of the protocol (days 7–10) as demonstrated by *APOB* (Figures 2E and 2F). These were apparent in Louvain clusters without expression of lung markers, namely clusters 0 and 12; while LPs seemed to be represented by clusters 1 and 10 (Figures S2E and S2F; Table S4). Finally, we performed gene category enrichment analysis to detect candidate pathways underlying the differentiation of lung progenitors. It showed an association between TGF-β/Smad and Wnt/β-catenin with the formation of DE, the Hippo pathway with FE differentiation, and Notch signaling in conjunction with insulin-like growth factor signaling with the NKX2-1+ lung progenitor cells (Figure 2G). Taken together, these results indicated that the specification of hepatoblasts originates at an earlier stage than FE formation and lung progenitors without requiring stimulation by exogenous FGF2, BMP4, or HGF.^17^^,^^18^

### Early transcriptional regulators of lung and hepatoblast specification

**Figure 3:**
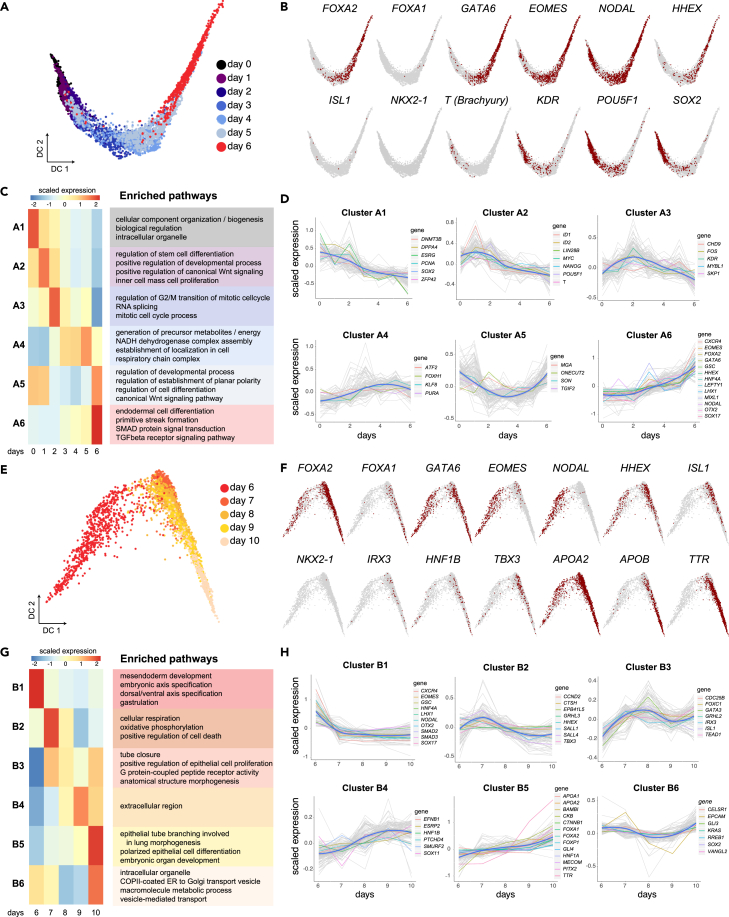
Dissection of the kinetic patterns of genes underlying the differentiation of lung progenitors and hepatoblasts (A and B) A diffusion map of single cells for days 0–6 showing the transition from pluripotency toward DE (A), with cells positive for indicated gene markers highlighted on top of the remaining cells (B). (C) Scaled mean expression per gene cluster and associated pathways identified by hierarchical clustering of the genes with significant associations to days 0–6 (adjusted p value <0.005, 6101 genes; high-to-low association colored red to blue). (D) Scaled mean expression of each gene cluster at the indicated time points. Gray lines correspond to the scaled expression values of the top 100 genes per cluster, and the blue lines correspond to the median expression within the cluster. (E–H) Diffusion map and cluster analysis as above for days 6–10 of differentiation (adjusted p value <0.005, 9352 genes). Genes discussed in the text are highlighted.

We next sought to identify genes and mechanisms that promote the emergence of lung progenitors, and the formation of liver cells at the DE stage, by focusing on the timing of up-regulation of known TFs. In this regard, *Isl1* has recently been characterized as a key regulator of *Nkx2-1* in the early FE,^40^ and *Hhex* has been associated with the activation of *Hnf4* TFs, which are essential for liver development.^41^ Moreover, the TFs *Gata6*, *Foxa1*, and *Foxa2* are well known for their crucial roles in the formation of the liver.^42^^,^^43^ Analysis of scRNA-seq data of the first 6 days of differentiation revealed the up-regulation of *GATA6*, *FOXA2*, and *HHEX*, but not of *ISL1* or *NKX2-1* (Figures 3A and 3B). Interestingly, the single cell data showed that only very few cells expressed *T (Brachyury)* in the first 6 days of differentiation (Figure 3B). Because the transient expression of *Brachyury*, followed by *Foxa2*, is known to occur in a subpopulation of the DE called mesendoderm,^20^ the absence of *T* indicated that the origin of the liver and lung cells is not associated with a mesendoderm origin. Collectively, this suggests that the regulation promoting the differentiation of liver cells is being established during the early specification of DE rather than upon FE formation.

To investigate further the circuitry of TFs that promotes DE specification, we used hierarchical clustering of all the genes that exhibited transcriptional changes during the first 6 days of differentiation. We generated six major clusters numbered A1-A6, representing different gene expression kinetics, and analyzed the enrichment of respective GO pathways in these clusters (Figures 3C and 3D; Table S6). Expression of major DE genes, such as *EOMES*, *LHX1*, *OTX2*, *CXCR4*, *LEFTY1*, and *SOX17* followed a very similar pattern with gradual upregulation peaking at day 6 (cluster A6). This cluster was enriched in pathways involved in endoderm differentiation and the regulation of SMADs and TGF-β signaling (Figure 3C; Table S6), including the loop of EOMES-Activin/Nodal signaling (Figure S3A). Several studies that have previously analyzed bulk DE cells have suggested that a subset of KDR+ (VEGF receptor 2) progenitors generates hepatic cells from differentiated human ESCs and that KDR signaling is important for their specification.^44^ Contrarily, we have noted that the up-regulation of *FOXA2*, *GATA4*, and *GATA6* coincided with a downward trend of KDR (Figure 3B and cluster A3 in d), suggesting that KDR is either not involved in instructing the early liver phenotype alongside lung cells or is only required in early differentiation phases.

To next investigate whether lung progenitors emerged during the second stage of the protocol, and whether this process coincided with maturation of hepatoblasts, we used hierarchical clustering to generate six clusters of gene expression, numbered B1–B6, for days 6–10 of differentiation (Figures 3E–3H; Table S6). We noted that the genes linked to the induction of DE, namely *EOMES*, *LHX1*, *GSC*, *OTX2*, *SOX17*, and *CXCR4*,^45^ and GO pathways associated with meso-endoderm development were sharply down-regulated upon dual-Smad inhibition in this stage (cluster B1, Figures 3G and 3H). An important exception was *FOXA2*, whose expression is essential for development of the foregut. *FOXA2* belonged to cluster B5 together with *FOXP1* and *PITX2* (Figure 3H), which are crucial for lung morphogenesis and asymmetry in the mouse, but not for the initial specification of the lung in the mouse.^36^^,^^46^ In accordance, GO pathways showed enrichment of lung morphogenesis, embryonic organ development and epithelial cell differentiation in cluster B5 (Figure 3G), even though at this stage *NKX2-1* had not been up-regulated yet (Figure 3F). Nevertheless, approximately halfway through this stage, *ISL1* was up-regulated in cluster B3 alongside *IRX3*. *Isl1* has recently been shown to regulate the development of lung lobes and trachea-esophagus tube separation by the activation of *Nkx2-1*,^40^ and *Irx3* is necessary for lung formation by promoting the proliferation of branched epithelium.^39^ In accordance, the GO patterns of cluster B3 included epithelial cell proliferation and tube closure, which collectively indicated that genes/hallmarks involved potentially in the initiation of the lung progenitor program had started to be regulated already within the FE stage (Figure 3G). *ISL1* is also known to regulate SHH in the FE,^47^ which suggested that basal differentiation of NKX2-1-eGFP+ cells was supported by endogenous production of SHH (Figure 1B). The connection to the activation of the SHH pathway at days 6–10 was apparent in clusters with an upward trend, including *GLI4* in B5 and *GLI3* in B6 (Figure 3H). Furthermore, comparison of our data with scRNA-seq of the mouse embryo at the time of gastrulation,^2^ showed that *Isl1* and *Irx3* were specifically enriched in the FE and the pharyngeal endoderm around E8.0-E8.5 (Figures S3B–S3E). In agreement with the FE association, pancreatic genes such as *PDX1*, which is associated with the midgut and suppressed by SHH,^48^ were not apparent in our data (Figure S3B).

Lastly, we analyzed hepatoblast and hepatocyte genes in days 6–10 following the activation of *HHEX* in days 0–6. At this stage we noted the upregulation of pioneering liver TFs including, *HNF1A*, *HNF1B*, and *TBX3* which governs the expansion of the liver bud,^49^ as well as first indications of functional liver genes, namely, *APOB*, *APOA2*, and Transthyretin (*TTR*) which are secreted by the liver (Figures 3F and 3H). Importantly, we noted that the expression of these liver genes was associated with Louvain clusters 0 and 12, which were to a large extent mutually exclusive from the aforementioned lung factors and related Louvain cluster 10 and 1 (Figures 2C and S2F; Table S4). Moreover, we analyzed the correspondence with scRNAseq data of human fetal liver tissues.^50^ We observed a significant association with gene signatures from human primary fetal hepatocytes, including *AFP*, *APOA1*, *F2*, *VNT*, *AGT*, and *APOB* in this time frame (Figure S5).

Taken together, these scRNA-seq data indicate that the lung and liver lineages are already set in their respective separate trajectories during the second stage of the protocol.

### Trajectory analysis reconstructs lung and liver branching

**Figure 4:**
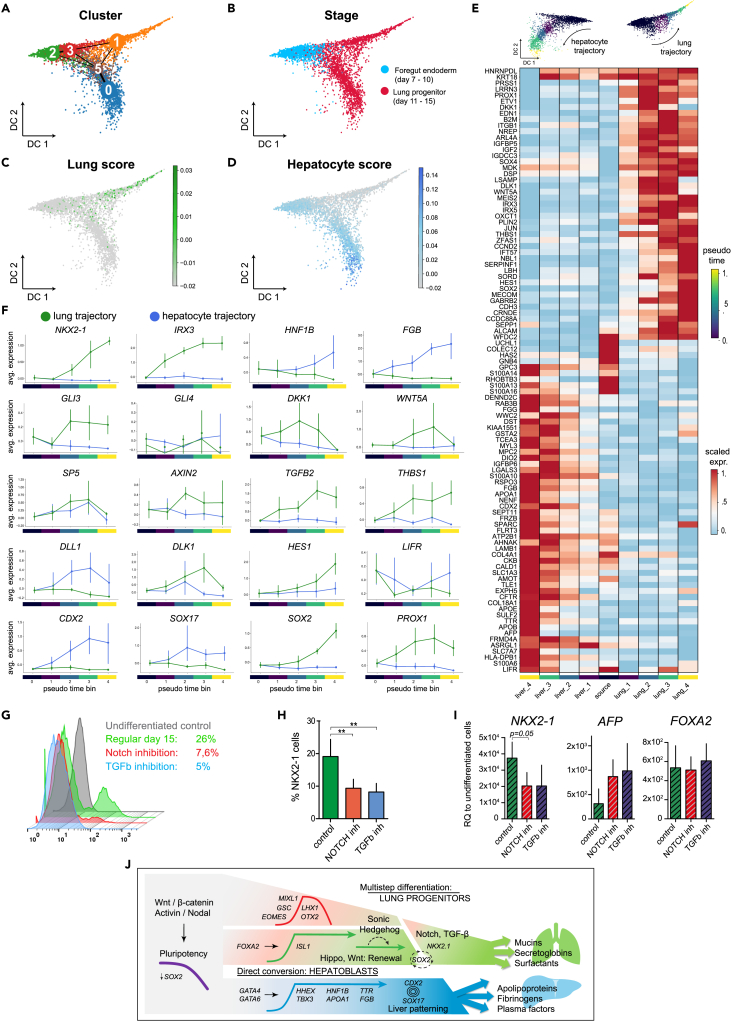
Reconstructing the transcriptional transitions from pluripotency to lung progenitors and hepatoblasts (A and B) Diffusion maps and Louvain clustering representing single cell transcriptomes of days 7–10 (blue) and 11–15 (red) in (B). (C and D) Color coded scores indicate the similarity of single cell transcriptomes to bulk mRNA-seq data of NKX2-1-eGFP+ (C) and eGFP- populations (D). (E) A heatmap showing unsupervised clustering of top differentially expressed genes along the lung and liver pseudotime trajectory, respectively. (F) Line plots showing the expression dynamics of the indicated genes in the respective branches in accordance with the binned pseudotime ordering (vertical lines represent confidence intervals of 95%). (G and H) Representative flow cytometry and the quantification of *NKX2-1-eGFP+* lung progenitors on day 15 of differentiation, with or without treatment by DAPT or SB431542 as indicated in days 11–15 (bars represent mean +SD, n = 4 biological replicates, ∗∗p < 0.01 by unpaired, one-tailed t-test). (I) The respective fold change of *NKX2-1, AFP*, and *FOXA2* on day 15 relative to the parental undifferentiated cells (quantified by RT-qPCR 2^(–ΔΔCT)^, bars represent mean +SD, n = 3 biological replicates, unpaired, one-tailed t-test). (J) A graphical summary of genes, their phases of expression, and mechanisms underlying the differentiation of lung progenitors and hepatoblasts from pluripotency state.

We next investigated the mechanisms that drive the separation of lung progenitors from the liver fate. Trajectory analysis of differentiation days 11–15, combined with days 7–10 revealed a branching event in the high dimensional single cell data manifold (Figures 4A, 4B, and S4). The lung progenitors appeared to become separated at the intersection of the FE stage with the last stage, and the maturity of lung and liver fates increased progressively over time when compared to NKX2-1eGFP+ and NKX2-1eGFP- bulk populations (Figures 4C and 4D). Importantly, the liver branch, but not the lung, exhibited an association with human fetal hepatocytes (Figure S5). As noted previously, the liver branch was established at days 7–10, preceding the appearance of a clear lung identity (Figures 4A and 4B).

The pseudotime trajectories in the last stage of the differentiation protocol revealed key differences in the lung and liver differentiation branches (Figures 4E and 4F). As expected, the expression of lineage specific markers such as *NKX2-1*, *IRX3*, and *HNF1B*, and *FGB* was exclusively increased along the two trajectories (Table S7). We noted that *GLI3*, a key transcriptional repressor that is regulated by SHH signaling, was upregulated only in the lung branch. This corresponded to the exogenous and endogenous activation of the pathway by SHH and *ISL1* (Figures 3G and 3H). Moreover, key components of the Wnt/β-catenin pathway, including *DKK1*, *WNT5A*, *SP5*, and notably the pathway’s canonical marker *AXIN2*, exhibited considerably higher expression in the lung trajectory (Figure 4F). The specificity of these pathways to the lung trajectory is particularly interesting given that exogenous treatment by SHH and CHIR (Figure 1A) did not activate the pathways in the neighboring hepatoblasts (Figure 1J). Considered together with earlier studies that revealed central roles for SHH and Wnt/β-catenin in foregut-early lung development,^30^^,^^51^^,^^52^ these results indicate that early hepatocytes might be refractory to these pathways. In accordance, the exclusive expression of *SOX2* in the lung trajectory is another indication of the lineage-specific activity of Wnt/β-catenin because SOX2, canonical Wnt signaling, and FGFs often intersect in the regulation of self-renewal in development (Figure 4F).^53^

Next, we found evidence for the activation of the Notch pathway specifically in the lung branch. This was deducted through the lung branch-specific expression of *HES1*, a canonical TF of the Notch pathway, as well as the Notch pathway modulator *DLK1* that is known to be involved in lung branching and morphogenesis.^54^ To experimentally determine if Notch is needed for lung specification in this system, we inhibited the Notch pathway using the γ-secretase inhibitor DAPT from day 11 onwards (Figures 4G–4I). In accordance with our hypothesis, this led to a significant decrease in the number of NKX2-1eGFP+ progenitors, a reduction in *NKX2-1* expression, and a reciprocal increase of *AFP* expression. Interestingly, a key ligand of the Notch pathway, namely *DLL1*, was specifically expressed in the liver branch (Figure 4F), suggesting that hepatoblasts might promote Notch signaling in lung progenitors by paracrine signaling. Moreover, we inhibited the TGF-β pathway using SB431542 after noting that the expression of *TGFB2* and *THBS1* was restricted to the lung branch (Figure 4F). We detected a similar effect to Notch pathway inhibition, namely, a decline in the number of NKX2-1-eGFP+ progenitors and expression of *NKX2-1* in treated cells (Figures 4G–4I). In summary, this suggests an important role for the interplay between Notch and TGF-β in the specification of the foregut toward lung progenitors.

Finally, the pseudotime trajectory ordering revealed the surprising involvement of key developmental genes that to our knowledge, have not been implicated in the specification of hepatoblasts and lung progenitors. We noted that *CDX2*, *SOX17*, and LIF/STAT3 signaling via *LIFR* expression were highly enriched specifically in the liver trajectory (Figure 4F), while mouse knockout studies have not repotred observations pertaining to the primary roles of these factors in liver development.^55^^,^^56^^,^^57^ Moreover, the expression of *PROX1* which is considered an early and specific marker for the developing liver and pancreas in the mouse FE,^58^ was in fact specifically expressed in the lung trajectory (Figure 4F). In addition, the expression of *FOXP1* and *PITX2* spanned both the lung and liver trajectories, and *FOXP2* was not detected (Figure S4E), contrary to the specific expression in lung progenitors described in the mouse embryo.^36^^,^^46^ This highlights differences in the regulation of key developmental genes between mouse and human, which we incorporated in a comprehensive roadmap model for human lung and hepatoblast differentiation from the pluripotency state (Figure 4J).

## Discussion

Lung disease is one of the leading causes of death. Lung organoid model systems based on the use of PSCs promise to greatly increase our ability to study lung disease. Likewise, harnessing the mechanisms of lung development will advance tissue engineering and regenerative medicine if we understand the fundamental differentiation processes during the formation of the lung and upon injury. Time-series single cell RNA-seq has been recently utlized to model the differentiation of human lung progenitors toward alveolar cell identities.^6^ In this study, we map even earlier molecular processes in human lung development by reconstructing genes, networks, and pathways during the transition from pluripotency to the NKX2-1 lung progenitor state.

Of key interest in the formation of lung progenitors is how the expression of *NKX2-1* is initiated in the foregut. Our analysis suggests the activity of FOXA2 and ISL1 in this process. We find that *FOXA2* is an epiblast-stage TF whose expression persists after the inhibition of TGF-β signaling in the second stage of the protocol, leading to abrupt down-regulation of *EOMES*, *MIXL1*, *GSC*. Interestingly, *FOXA2* has been demostrated to directly activate *ISL1* in cardiac progenitors,^59^ and *ISL1* has been reported to activate *NKX2-1* in the early FE in a process essential for lung formation.^40^ Moreover, Shh signaling is necessary for the development of the FE,^30^^,^^31^ ISL1 is known to regulate *Shh* expression in the FE,^47^ and here we demostrate that SHH treatment enhances the number of NKX2-1 progenitors. The link between ISL1 and SHH signaling is further suported by the similar developmental defects of the cardiovascular system in *Isl1* and *Smo* (*Smoothened*) mutant mouse embryos.^47^

Importantly, complexes of Foxa2, Otx2, and Lhx1 have been implicated in autoregulation and in pioneering the transcription of FE related genes.^45^ Based on the similar expression kinetics of *FOXA2* and *OTX2* and the up-regulation of *LHX1* observed in our data, we reason that a similar process might control the progenitor differentiation of lungs *in vitro*. This is illustrated in our prposed model which posits FOXA2 and OTX2 in an autoregulatory loop during the formation of early DE, inducing the expression of ISL1 and subsequently NKX2-1. Concurrently ISL1 is postulated to up-regulate and activates SHH signaling which *in vivo* has been shown to promote lung morphogenesis and branching patterns (Figure 4J).^31^^,^^60^

The emergence of both lung and liver cells in the differentiation protocol posed the question if a common progenitor can be identified. Based on our trajectory analysis we found no evidence indicating the formation of bipotent lung-liver progenitors. We found that the inception of hepatoblast transcription networks occurs much earlier than the initiation of the lung program. The induction of *HHEX*, *HNF4A*, and *HNF1B* are unmistakable signs of hepatoblast differentiation, and remarkably these genes were up-regulated as early as 3–4 days of DE differentiation, while the earliest signs of *ISL1* upregulation did not appear until 4–5 days later. Therefore a mechanism whereby a common progenitor population responds to cues that promote a choice between alternate fates, specifically lung or liver, is unlikely in this context. While we have not detected a strong signal for T-positive mesendoderm intermediate progenitors, we do not exclude the possibility, as scRNA-seq may not fully capture TFs with low expression levels.

Our data indicate a direct conversion of early DE to hepatoblasts. Mechanistic mouse studies show that the activation of the Activin/Nodal—EOMES loop initiates on the high levels of canonical TFs that control hepatogenesis in the mouse embryo. This includes *Gata6* and *Gata4*,^43^ *Foxa2*, and *Foxa1*,^61^ which have been implicated in pioneering chromatin remodeling in FE precursors,^62^ and were utilized in the induction of hepatocyte transdifferentiation.^63^^,^^64^ Moreover, *Gata4* and *Gata6* have been shown to be functionally redundant in promoting the development of the liver, and together with *Foxa2* they promote liver specification by inducing *Hhex*.^65^ Remarkably, in our data a number of hepatoblast TFs, such as *HNF4A*, *TBX3*, and liver factors that are secreted into the plasma, such as *APOA2*, *FGB*, *TTR*, *APOB* were induced shortly after *HHEX.* Also, the gene signature that was activated in this stage in the liver branch showed the highest correspondence with fetal hepatocytes when compared to human liver development (Figure S4). Collectively, this supports our “fast track” liver differentiation model from early DE (Figure 4J). Interestingly, the expression of *HHEX* itself ceased almost entirely in the third phase of the protocol, despite the continued expression of *FOXA2* and *GATA6*. It is possible that a majority of cells had undergone specification already at the beginning of the third phase of the protocol, or that the disappearance of *HHEX* is connected to the downregulation of *OTX2*, which directly regulates the *HHEX* promoter FE cells,^66^ or that a different mechanism is in play.

Of special interest for tissue engineering and disease modeling is the association of the *FOXA2* - *ISL1* - *NKX2-1* trajectory with developmental pathways that regulate patterning and self-renewal in the lung (Figure 4J). Our work highlights (i) the up-regulation of *SOX2* in the lung branch that coincided with the activation of Notch and Wnt/β-catenin signaling, (ii) the potential involvement of the Hippo pathway during the foregut stage of the differentiation protocol, (iii) the reduction of NKX2-1 progenitors upon TGF-β and Notch inhibition, together with (iv) Yap-dependent TGF-β-mediated induction of proliferation by Sox2 in mouse airway progenitors,^67^ creating a putative picture of renewal mechanisms that might inform the future derivation of human lung stem cells. Accordingly, Ikonomou et al.^68^ demonstrated the upregulation of Hippo and TGF-β activity prior to and upon early lung specification, respectively. Moreover, the specific expression of *SOX17* and *CDX2* in the liver trajectory is novel and important. In the mouse *CDX2* expression is restricted to the hindgut where it serves to regulate intestinal stem cell development.^55^ Therefore, the up-regulation of *CDX2* in conjunction with *SOX17*, which serves as a patterning factor in the liver bud,^56^ might indicate that these factors play central roles in the regulation of human liver development. Comparing the trajectories of additional foregut-lung TFs with their associated functions in mouse development identified additional differences. Specifically, we noted the broadening of the expression profiles of *FOXP2* and *PITX2*, which might imply that evolutionary changes have increased their involvement in the differentiation of the human foregut. Another example is the association of *PROX1* with lung progenitors as opposed to hepatocytes in the mouse.^44^ This reveals putative mechanisms that are specific to the formation of human FE precursors and their differentiation into lung and liver progenitors.

In summary, the high temporal resolution of our single cell trajectory analysis enabled us to construct a hierarchical model of gene expression changes along two trajectories from hPSC to lung and liver fate, respectively. In our analysis the pseudotime estimation aligned with the highly resolved time series of sampling. Interpreting our results in light of *in vivo* studies provided a detailed understanding of mechanisms, their timing, and evolutionary changes that regulate the specification of lung progenitors in the foregut. These findings should inform applications of iPSCs in tissue engineering efforts for regenerative medicine of the lung, possibly including the derivation of lung stem cells. Moreover, our work offers in parallel a high resolution “roadmap” for a potential direct conversion of hepatoblasts, contributing to the improvement of the required knowledge for manufacturing of hepatocyte grafts in liver disease.

### Limitations of the study

In this study, we used a high temporal resolution of sampling to delineate gene expression trajectories in the differentiation of hPSC toward lung progenitors. The resulting pseudo-temporal model of a lung and liver trajectory requires further dissection using functional perturbations to distinguish between correlation and causation. We have addressed the functional role of Notch and TGF-beta signaling as well as SHH in the formation of lung progenitors. Based on our data, we cannot conclude the mechanism by which SHH improves the differentiation efficiency of lung progenitor cells. A promising direction for future research would be to explore the autocrine function of SHH signaling in early lung development.^60^ Furthermore, a previous study has also demostrated the beneficial role of SHH during FE generation from PSCs.^69^ Interestingly, despite the established regulatory role of FGF10 in lung development *in vivo*, studies have shown that FGF10 is not necessary during the *in vitro* differentiation of PSCs into LPs.^13^^,^^14^^,^^15^^,^^16^ In our system, FGF10 appears to facilitate the differentiation outcome in the absence of SHH despite its dispensable role. Hence, it would be relevant to further investigate this complex web of cytokine interactions in order to delineate mechanisms stemming from the (epi)genetic differences between PSC lines, which might influence their differentiation efficiencies.

## STAR★Methods

### Key resources table

**Table:** 

REAGENT or RESOURCE	SOURCE	IDENTIFIER
**Antibodies**		

Anti-FOXA2	Abcam	Cat# ab60721; RRID: AB_941632
Anti-proSP-C	Millipore	Cat# AB3786; RRID: AB_91588
Anti-pro and mature SP-B	Abcam	Cat# ab40876; RRID: AB_778186
Anti-SCGB1A1	Santa Cruz Biotechnology	Cat# sc-365992; RRID: AB_10915481
Anti-Mucin 5AC	Abcam	Cat# ab79082; RRID: AB_1603327
Anti-AFP	Sigma Aldrich	Cat# WH0000174M1; RRID: AB_1839587
Anti-EpCAM APC	BD Biosciences	Cat# 347200; RRID: AB_400570
Anti-c-KIT APC	ThermoFisher Scientific	Cat# CD11705; RRID: AB_2536476
Anti-CXCR4 PE	ThermoFisher Scientific	Cat# MHCXCR404; RRID: AB_10373097
goat anti-mouse Alexa Fluor™ 488	ThermoFisher Scientific	Cat# A-11001; RRID: AB_2534069
goat anti-rabbit Alexa Fluor™ 647	ThermoFisher Scientific	Cat# A-21244; RRID: AB_2535812
goat anti-rabbit Alexa Fluor™ 546	ThermoFisher Scientific	Cat# A-11035; RRID: AB_2534093
donkey anti-mouse Alexa Fluor™ 594	ThermoFisher Scientific	Cat# A-21203; RRID: AB_141633
mouse IgG2a kappa isotype control, PE	ThermoFisher Scientific	Cat# 12-4724-41; RRID: AB_1603328
mouse IgG2a kappa isotype control, APC	ThermoFisher Scientific	Cat# 17-4724-81; RRID: AB_470188

**Chemicals, peptides, and recombinant proteins**		

Recombinant Activin A	R&D Systems	Cat# 338-AC
Ascorbic Acid	Sigma Aldrich	Cat# A8960-5G
Recombinant Human BMP4	R&D Systems	Cat# 314-BP
B-27™ Supplement	ThermoFisher Scientific	Cat# 17504044
BSA (Bovine Albumin Fraction V (7.5% solution))	ThermoFisher Scientific	Cat# 15260037
8-bromo-cAMP (8-Bromoadenosine 3,5-cyclic monophosphate)	Sigma Aldrich	Cat# B5386
CHIR99021	R&D Systems	Cat# 4953/50
DAPT	TOCRIS	Cat# 2634
Dexamethasone	Sigma Aldrich	Cat# D4902
DMEM/F12	ThermoFisher Scientific	Cat# 11320033
Dorsomorphin dihydrochloride	TOCRIS	Cat# 3093
Recombinant Human FGF-7 (KGF)	R&D Systems	Cat# 251-KG
Recombinant Human FGF-10	R&D Systems	Cat# 345-FG
GlutaMAX™ Supplement	ThermoFisher Scientific	Cat# 35050038
Ham's F-12 Nutrient Mix	ThermoFisher Scientific	Cat# 21765029
IBMX (3-Isobutyl-1-methylxanthine)	Sigma Aldrich	Cat# I5879
IMDM	ThermoFisher Scientific	Cat# 12440053
1-Thioglycerol	Sigma Aldrich	Cat# M6145
Corning® Matrigel® Growth Factor Reduced (GFR) Basement Membrane Matrix	Corning	Cat# 354230
N-2 Supplement	ThermoFisher Scientific	Cat# 17502048
Penicillin-Streptomycin	ThermoFisher Scientific	Cat# 15070063
Retinoic Acid	Sigma Aldrich	Cat# R2625
RPMI 1640 Medium	ThermoFisher Scientific	Cat# 21875034
SB431542	Miltenyi Biotec	Cat# 130-106-543
Recombinant Human SHH	R&D Systems	Cat# 1845-SH
Sodium Butyrate	Sigma Aldrich	Cat# B5887
Y-27632 dihydrochloride	R&D Systems	Cat# 1254

**Critical commercial assays**		

QuantSeq 3′ mRNA-Seq Library Prep Kit for Illumina (REV)	Lexogen	Cat# 015.24
RNeasy Mini Kit	Qiagen	Cat# 74106
Verso cDNA synthesis kit	ThermoFisher Scientific	Cat# AB1453A
Power SYBR® Green PCR Master Mix	ThermoFisher Scientific	Cat# 4367659
High Sensitivity DNA Kit	Agilent	Cat# 5067-4626

**Deposited data**		

scRNA-seq raw and analyzed data	This paper	GEO: GSE167011
Analysis code	This paper	https://github.com/theislab/2020_iPS_Lung_Differentiation

**Experimental models: Cell lines**		

*NKX2-1*^*eGFP/+*^		Olmer et al.^32^

**Oligonucleotides**		

*FOXA2* F: GGGAGCGGTGAAGATGGA R: TCATGTTGCTCACGGAGGAGTA		
*SOX17* F: GGCGCAGCAGAATCCAGA R: CCACGACTTGCCCAGCAT		
*CXCR4* F: CACCGCATCTGGAGAACCA R:GCCCATTTCCTCGGTGTAGTT		
*NKX2-1* F: CTTCCCCGCCATCTCCCGCTTC∗ R: GCCGACAGGTACTTCTGTTGCTTG	∗Reverse (R) Primer from^12^	
*PAX8∗* F: ACTACAAACGCCAGAACCCTACCA R: CCGGATGATTCTATTAATGGAG	∗Forward (F) Primer from^12^	
*PAX6* F: GCGGAGTTATGATACCTACACC R: GAAATGAGTCCTGTTGAAGTGG		
*MUC5AC* F: GCCTACGAGGATTTTAACAT R: CAGGACCGGGTGGCCGTTGA		
*SFTPC* F: GTTCTGGAGATGAGCATTGGG R: GCGATCAGCAGCTGCTGGTAGT		
*SFTPB* F: CTGGCCCAAGGTCGCCGA∗ R: TGGAGCATTGCCTGTGGTATGG	∗Reverse (R) Primer from^12^	
*SCGB1A1* F: ATGGACACACCCTCCAGTTATG R: TGGGCTATTTTTTCCATGAGC		
*AFP* F: GCTTACACAAAGAAAGCCC R: TAATAATGTCAGCCGCTCC		
*GAPDH* F: GCTCATTTCCTGGTATGACAACG R: GAGATTCAGTGTGGTGGGGG		
Smart PCR Primer: AAGCAGTGGTATCAACGCAGAGT		

**Software and algorithms**		

BD FACSDiva Software		
GraphPad Prism 7.0	GraphPad	
FlowJo software		
FIJI (ImageJ) software	National Institutes of Health	
String platform	Szklarczyk et al.^70^	
Panther	Thomas et al.^71^	
SeqMonk	https://www.bioinformatics.babraham.ac.uk/projects/seqmonk	v1.45.4
corrplot	Simko and Wei^72^	v0.84
diffxpy	https://github.com/theislab/diffxpy	v0.18
Dropseq-tools	https://github.com/broadinstitute/Drop-seq	v2.0.0
ggplot2	Wickham et al.^73^	v3.2.1
limma	Ritchie et al.^74^	v3.48
pheatmap	Kolde et al.^75^	v1.0.12
python	https://www.python.org	v3.8
R	http://www.r-project.org	v3.6.1
scanpy	Wolf et al.^76^	v1.6.1
scvelo	Bergen et al.^77^	v0.2.5
Seurat	Satija et al.^78^	v2.3
STAR	Dobin et al.^79^	2.5.2a

### Resource availability

#### Lead contact

Further information and requests for resources and reagents should be directed to and will be fulfilled by the lead contact, Micha Drukker (m.drukker@lacdr.leidenuniv.nl).

#### Materials availability

This study did not generate new unique reagents.

### Experimental model and study participant details

#### Stem cell maintenance and passaging

Human iPSC line *NKX2-1*^*eGFP/+*^ was kindly provided from Hannover Medical School,^32^ maintained in feeder-free conditions, in StemMACS iPS-Brew XF (Miltenyi Biotech) and passaged with Accutase (Sigma-Aldrich) on tissue cell culture plates pre-coated with 1:100 dilution of Geltrex Basement Membrane Matrix in DMEM/F-12 (both from ThermoFisher Scientific).

### Method details

#### Differentiation of hiPSCs into NKX2-1+ early lung progenitors

To induce DE differentiation, hiPSCs were maintained in iPSC-Brew and when reached 80% confluency (day 0), the cells were rinsed with DPBS and incubated in Accutase (Sigma- Aldrich) for 10 minutes, at 37°C. The detached cells were triturated into single-cell suspensions and seeded onto 24-well plates pre-coated with 1:40 Growth Factor Reduced (GFR) Matrigel (Corning), in a density of 2 x 10^5^ cells/cm^2^. Cells were immediately treated with 100 ng/ml Activin-A, 1 μM CHIR99021, and 10 μM Y-27632 (all from R&D Systems), in Definitive Endoderm Basal Media (DE-BM) consisting of RPMI1640 medium, 1 x B27 supplement, 1 x NEAA and GlutaMAX (all from ThermoFisher Scientific). On days 1- 6 the DE-BM was supplemented with 100 ng/ml Activin-A, 1 μM CHIR99021 (R&D Systems) and 0.25mM (day 1) and 0.125 mM (days 2-6) sodium butyrate (Sigma-Aldrich).^15^

For the FE stage the Basal Mediums (FE-BM1 and FE-BM2) tested, were prepared as follows, FE-BM1: DMEM/F12, 1 x GlutaMAX, 1 x B-27 and N-2 supplements, 50 U/ml of penicillin/streptomycin, 0.05 mg/ml of L-ascorbic acid (Sigma-Aldrich) and 0.4 mM of 1-thioglycerol (Sigma-Aldrich)^15^ or FE-BM2: 75% IMDM, 25% Ham’s F-12, 0.5 x B27 supplement and N2 supplement, 0.05% bovine serum albumin, 1 x GlutaMAX, 50 U/ml of penicillin/streptomycin (all from ThermoFisher Scientific), 0.05 mg/ml of L-ascorbic acid and 0.4 mM of 1-thioglycerol (Sigma-Aldrich)^10^ (Figure 1B). On day 6 DE cells were collected with Accutase for 10 minutes at 37°C and re-plated in a density of 1:2-1:4 onto GFR Matrigel-coated plates. The cells were treated for 2 days (days 6, 7) with 50 ng/ml sonic hedgehog (SHH; R&D Systems), 2 μM dorsomorphin (DSM; Tocris) and 10 μΜ SB431542 (Miltenyi Biotec), supplemented with 10 μM Y-27632 (R&D Systems) for the first 24 hours. On day 8 the medium was changed to FE-BM1 or 2 respectively, with 2 μM DSM and 10 μΜ SB431542.

To induce the lung progenitor (LP) stage, on the day 10, the medium was switched to FE-BM1 or BM2 containing 20 ng/ml recombinant human BMP4 (rhBMP4, R&D Systems), 50 nM retinoic acid (RA; Sigma-Aldrich), 3 μΜ CHIR99021 and 20 ng/ml rhFGF10 (R&D Systems). For the inhibition of Notch and TGF-β pathways, 10 μΜ SB431542 and 100 μM DAPT (TOCRIS Bioscience) were additionally used at the LP induction stage of differentiation respectively. Growth media and supplements are listed on Table S1.

#### Flow cytometry analysis and sorting of iPSC-derived lung progenitors

The cells were rinsed with DPBS and incubated in Gentle Cell Dissociation medium (STEMCELL Technologies)/0,05%Trypsin (ThermoFisher Scientific) (2vol:1vol) for 10 min, at 37°C. The detached cells were diluted in a FACS buffer containing 2% FBS/DPBS and centrifuged at 200 RCF for 3 minutes, at room temperature (RT). Cell pellets were washed with FACS buffer and centrifuged again. For analysis, the samples were incubated (if necessary) with the conjugated antibodies diluted in FACS buffer, for 25 min on ice, washed twice with FACS buffer and analyzed. Live cells were distinguished by staining with propidium iodide (PI) or Sytox blue (SB). For sorting, the dissociated cells were resuspended in FACS buffer, stained with PI and sorted into 2% FBS/DMEM. The measurements were obtained using a BD FACSAria III flow cytometer (BD Biosciences). Isotype controls were used for gating stained cells, whereas undifferentiated cells that do not express eGFP were used as negative control for gating NKX2-1^eGFP+^ cells.

#### Generation of 3D cultured lung organoids

On day 15 of the 2D differentiation, the cells were rinsed with DPBS and the eGFP+ colonies were dissected with a needle, picked and transferred to 80% Matrigel (diluted in FE-BM2). Drops of 40μL were pipetted into the center of each well of a 24 well tissue culture plate and incubated for 30 min at 37°C. Then, FE-BM2 media supplemented with 10 ng/ml FGF10, 10 ng/ml FGF7 and 3 μM CHIR99021 was added to each well for 7 days.^10^ On day 22 the medium was switched to FE-BM2 media supplemented with 50 nM dexamethasone (Sigma-Aldrich), 0.1 mM 3-Isobutyl-1-methylxanthine (IBMX; Sigma-Aldrich), 0.1 mM 8-Bromoadenosine 30,50-cyclic monophosphate sodium salt (cAMP; Sigma-Aldrich)^8^ and ± 10 μM SB431542, 3 μM CHIR99021. For all the 3D Matrigel culture conditions the medium was changed every second day. Growth media and supplements are listed on Table S1.

#### Immunostaining

For imaging 2D cultures cells were cultivated on removable 8-well chamber glass slides (Ibidi), rinsed twice with DPBS and fixed with 4% PFA (Sigma-Aldrich)/DPBS for 20 min at RT. Cells were permeabilized with 0.2% Triton X-100 (ThermoFisher Scientific) for 5 min and blocked with 3% BSA for 30 min at RT. Then, cells were incubated with primary antibodies diluted in solution containing 0.1% Triton X-100/3% BSA overnight at 4°C, followed by three washes with DPBS, after which they were incubated with secondary antibody in 0.1% Triton X-100/3% BSA solution for 1 hour at RT and washed three times with DPBS. The removable wells were discarded and ProLong^TM^ Gold Antifade Mountant with DAPI (Thermo Fisher Scientific) was used to mount the coverslip.

For imaging lung organoids, the samples were extracted from the Matrigel drops, upon incubation with 2mg/ml Dispase IV (Sigma-Aldrich), at 37°C for 1h. Then, the organoids were fixed with 4% paraformaldehyde/DPBS for 2 hours at 4°C and incubated in 30% sucrose/DPBS overnight at 4°C. The samples were embedded in OCT compound (Sakura Finetek) and frozen in -20°C. The frozen samples were cryosectioned into 6-μm slices, permeabilized and blocked with 0.5% Triton X-100/5% Normal Goat or Donkey Serum (NGS or NDS) for 30 min in RT. Next, the slices were incubated with the primary antibodies, diluted in 0.5% Triton X-100/5% NGS or NDS, overnight at 4°C, followed by washing three times with DPBS (10 min each), and stained with secondary antibodies for 3h at RT. After washing twice more, a coverslip was mounted using ProLong^TM^ Gold Antifade Mountant with DAPI. All antibodies used for the study are listed on Table S1.

#### Quantitative RT-PCR

For the RT-qPCR analysis, the cells were lysed and subsequent RNA extraction was performed using the RNeasy Mini Kit (Qiagen) according to manufacturer’s instructions. The RNA was reverse transcribed into cDNA using the Verso cDNA Synthesis Kit (ThermoFisher Scientific) and RT-qPCR was performed in 384-well plates using the Power SYBR Green Master Mix (ThermoFisher Scientific) in a total reaction volume of 10 μl, using a QuantStudio 12K Flex qPCR machine (ThermoFisher Scientific). Following cycling conditions were applied: 2 min at 50°C, 10 min at 95°C, 44 x 15 sec at 95°C and 1 min at 60°C. Primers are listed on Table S1.

#### Bulk mRNA-seq library construction and sequencing

Transcriptome analysis of undifferentiated iPSCs (day 0), sorted NKX2-1 eGFP+ and NKX2-1 eGFP- (day 15) and DCI(±CS)-treated LOs (day 35) was carried out by using the QuantSeq 3′ mRNA-Seq Library Prep Kit for Illumina (REV) with Custom Sequencing Primer (Lexogen) according to manufacturer’s instructions. The 3′ mRNA sequencing libraries were prepared from 110 ng of total input RNA per sample which was isolated with the RNeasy Mini (QIAGEN). Libraries were amplified and multiplexed with barcodes under the following conditions: 98°C 30 sec, 14 cycles of 98°C for 10 sec, 65°C for 20 sec, 72°C for 30 sec, and a final extension of 72°C for 1 min. Quality of the libraries was evaluated on a Agilent 2100 Bioanalyzer using the High Sensitivity DNA Kit (Agilent Technologies). Libraries were denatured with 0.1 N NaOH, diluted to a final concentration of 6 pM and samples were sequenced using HiSeq2500 machine. Analyses were performed using SeqMonk (Babraham Bioinformatics, https://www.bioinformatics.babraham.ac.uk/projects/seqmonk). Differential expression analysis was performed with DESeq2 within SeqMonk. GO terms analysis was performed using Panther^71^ and STRING platforms.^70^

#### Single cell RNA-seq (Droplet-sequencing)

##### Generation of single-cell suspensions

Daily sampling of cells during differentiation was performed by detaching the cells from the tissue culture plate using Accutase. Cells where thereafter centrifuged for 5 min at 300 × *g* (4°C), counted using a Neubauer chamber and critically assessed for single-cell separation and viability. A total of 250,000 cells were aliquoted in 2.5 ml of PBS supplemented with 0.04% of bovine serum albumin (BSA) and loaded for DropSeq at a final concentration of 100 cells/μl.

##### Single-cell RNA sequencing

Drop-seq experiments were performed according to the original Drop-seq protocol.^37^^,^^80^ Using a microfluidic polydimethylsiloxane device (Nanoshift) single cell (100/μl) suspensions were co-encapsulated in droplets with barcoded beads (120b/μl, purchased from ChemGenes Corporation, Wilmington, MA) at rates of 4000 μl/h. Droplet emulsions were collected for 15 min/each prior to droplet breakage by perfluorooctanol (Sigma-Aldrich). After breakage, beads were harvested and the hybridized mRNA transcripts reverse transcribed (Maxima RT, Thermo Fisher). Unused primers were removed by the addition of exonuclease I (New England Biolabs), following which beads were washed, counted, and aliquoted for pre-amplification (2000 beads/reaction, equals ∼100 cells/reaction) using a total of 10 PCR cycles. PCR details: (Smart PCR primer: AAGCAGTGGTATCAACGCAGAGT (100 μM), 2× KAPA HiFi Hotstart Readymix (KAPA Biosystems), cycle conditions: 3 min 95°C, 4 cycles of 20 sec 98°C, 45 sec 65°C, 3 min 72°C, followed by 8 cycles of 20 sec 98°C, 20 sec 67°C, 3 min 72°C, then 5 min at 72°C).^37^

PCR products of each sample were pooled and purified twice by 0.6 × clean-up beads (CleanNA), following the manufacturer’s instructions. Prior to tagmentation, complementary DNA (cDNA) samples were loaded on a DNA High Sensitivity Chip on the 2100 Bioanalyzer (Agilent) to ensure transcript integrity, purity, and amount. For each sample, 1 ng of pre-amplified cDNA from an estimated 600 cells was tagmented by Nextera XT (Illumina) with a custom P5 primer (Integrated DNA Technologies). Single-cell libraries were sequenced in a 100 bp paired-end run on the Illumina HiSeq4000 using 0.2 nM denatured sample and 5% PhiX spike-in. For priming of read 1, 0.5 μM Read1CustSeqB was used (primer sequence: GCCTGTCCGCGGAAGCAGTGGTATCAACGCAGAGTAC).

#### Bioinformatics Processing of the data set

The count matrices for each sample were generated by the Drop-seq computational pipeline (version 2.0) as previously described.^37^ Briefly, STAR (version 2.5.2a) was used for mapping.^79^ Reads were aligned to the hg19 reference genome (provided by the Drop-seq group, GSE63269). During downstream analyses we employed the Seurat package (v2.3, Satija et al., 2015) for pre-processing up to the visualization and Louvain clustering of the full data set, while the Scanpy package (v1.6.1)^76^ was used for PAGA construction and analyses/visualization of the individual stage subsets. For barcode filtering, we excluded barcodes with less than 400 detected genes. As 1000 cells were expected per sample, the first 1200 cells, sorted by number of transcripts per cell, were used further. As a high proportion of transcript counts derived from mitochondria-encoded genes may indicate dying or stressed cells, we removed cells with a percentage of mitochondrial genes of above 20% from downstream analysis. Guided by histograms of quality metrics, further filtering was carried out. Cells with a high number of UMI counts may represent doublets, thus only cells with less than 5000 UMIs were used in downstream analysis. Quality metrics for each sample can be inspected in Table S2.

After the initial filtering of cells, we followed the common preprocessing procedure. The expression matrices were normalized and scaled using Seurat’s NormalizeData() and ScaleData() functions. In order to mitigate the effects of unwanted sources of cell-to-cell variation, cell-cycle effects, percentage of mitochondrial reads and number of counts were regressed out CellCycleScoring() and ScaleData(). The 600 top variable genes across the data set were selected via FindVariableGenes() using the default parameters. From this list the known cell cycle genes were excluded. These variable genes were then the basis of the principal component analysis. The first 50 Components were the input for Seurat’s function FindClusters() at a resolution of 2, resulting in 13 clusters. To visualize the clustering result of the high dimensional single-cell data, the UMAP was generated using again 50 components as input for the Seurat function RunUMAP() with a number of neighbors set to 20. To clean up the data set further, a small number of cells disagreeing in the louvain cluster and UMAP embedding were removed. The final meta data and marker gene expression per cluster is reported in Tables S3 and S4, respectively.

##### Selection of genes with significant association over time

To account for the harsh perturbation in gene expression induced by the medium change, the consequent analyses were split into three stages corresponding to the definite endoderm DE (day 0 to day 6), foregut endoderm FE (day 7 to day 10) and lung progenitors LP (day 11 to day 15). The stage wise temporal analyses were executed using the python package Scanpy.^76^

The principal components and the neighborhood graph based on the first 10 components was recalculated for each stage separately using Scanpy’s pp.pca() and pp.neighbors(). After generating the diffusion maps via the tl.diffmap() function setting the root cells after manual inspection, the diffusion pseudotime was calculated with tl.dpt(). The diffusion pseudotime for the whole data set was then set by adding the stage-wise pseudotimes and scaled to a value between 0 and 1. To assess global connectivity, the louvain clusters were used as input for Partition-based graph abstraction (PAGA) tl.paga(). The weighted edges reflect a statistical measure of connectivity between the groups. Edges with weight < 0.05 are omitted.

For the identification of genes showing significantly changing expression patterns over time, the following approach was applied by the means of the R packages limma^74^ and splines. As we were particularly interested in the expression pattern towards early lung progenitors, we selected only those cells that were positive for either eGFP or NKX2-1 from days 11 to 15 for the calculation and display. Furthermore, as we did not want to incorporate differences in ribosomal derived genes, such genes were excluded from this analysis. We employed a regression model in which splines are used to model non-linear effects of continuous variables to fit the time-course data. For each gene we fit a natural cubic spline with 4 knots while using the time point of extraction as an explanatory variable. UMI counts of each cell were included as a covariate in the model to account for differences in library size.

We rank the genes based on their adjusted p-values. As it is non-trivial to interpret p-values across time, we use the adjusted p-value as a ranking for the genes and visualize the top 200 genes in Figure 2E. The results are reported in Table S5.

##### Stage-wise hierarchical clustering of genes

The clustering analysis was done for the endoderm stage (day 0 to day 6) and the combined endoderm and FE stage (day 0 to day 10). For this, genes for both versions with interesting expression patterns were again selected as described previously using the regression model based on spline fits. The scaled expression levels of genes with an adjusted p-value of less than 0.005 were the input for hierarchical clustering using the hdist() from the R package stats. The dendrogram tree was cut into 10 clusters, which were manually re-annotated into 6 final groups for each stage based on their average expression per day. The cluster sizes can be different, thus we chose to display the average expression of the 100 genes with the lowest adjusted p-value per cluster in Figures 3D and 3H. For the second clustering analyses, we display the average expression and kinetic profiles for day 6 to day 10. The resulting clusters are reported in Table S6.

##### Potential branching into lung and non-lung progenitor cells

To identify genes associated with the potential differentiation branches, we used the signatures from the differential gene expression analysis between eGFP labeled cells versus eGFP negative cells in the initial bulk experiment. As variable genes those genes were selected, which showed a log fold change of above 1 and below -1. To reduce the effect of the media change after day 10, we removed genes that were differentially expressed between the FE and LP stage. In order to guide the dimensionality reduction these 1294 genes were used as input for the PCA. Following Scanpy’s workflow, the knn graph was constructed via pp.neighbors(n_neighbors = 10, n_pcs = 50), and the UMAPs and diffusion maps were generated via tl.umap() and tl.diffmap(), respectively. In the next step we filtered for the two branches of interest, which started in the FE stage and had their endpoint in the LP stage. The cells were scored to their similarity to the bulk signature via tl.score(), to evaluate if the two potential cell fate trajectories correspond to the branches that we found.

To identify genes showing temporally altered expression patterns along these two potential branches, which are also significantly different across the branches, we employed the python package diffxpy (https://github.com/theislab/diffxpy). The starting population for the two sub-branches (lung and hepatocyte progenitors) was chosen as all cells from FE stage for both trajectories. The cells from the DE stage were divided based on their louvain clusters after visual inspection of the diffusion map and known lineage marker gene expression. The diffusion pseudotime was calculated on these two trajectories separately. Diffxpy’s test.continuous_1d() function runs the test with a spline basis to allow smooth trends. This function was employed on the combination of these two trajectories, using pseudotime as a continuous covariate and a categorical annotation “trajectory” (implying which branch each cell was assigned to) as factor to test for. For visualization purposes, the trajectory-wise pseudotimes were manually binned into “source” and 4 groups each. Average expression of cells per bin is shown for top 100 genes ranked by adjusted p-value from the diffxpy result table in Figure 4E. The results are reported in Table S7.

##### Pairwise gene expression profiles along pseudotime

Cells ordered by diffusion pseudotime were partitioned into 9 bins for the liver and 10 bins for the lung branch similarly as described above. Pairwise gene correlations across the two trajectories were calculated with the cor() function (default parameters, R version 3.6.1) using the average expression in each bin, and visualized using corrplot package in R (version 0.84).

### Quantification and statistical analysis

Statistical analyses of the Bulk mRNA-seq were performed using SeqMonk. Differential expression analysis was performed with DESeq2 within SeqMonk. GO terms analysis was performed using Panther^71^ and STRING platforms.^70^ Statistical analysis and functional enrichment for the generated scRNA-seq data are described in detail in the above ‘‘Bioinformatics Processing of the data set’’ section. Sample number (n), replicates and statistical method used are described in each figure legend.

## Data Availability

• The scRNA-seq data used in this study has been deposited at GEO and are publicly available as of the date of publication. Accession numbers are listed in the key resources table. • All original code has been deposited at GitHub and is publicly available as of the date of publication. The link is listed in the key resources table. • Any additional information required to reanalyze the data reported in this paper is available from the lead contact upon request.

## Supplemental information

Document S1. Figures S1–S5 and Table S2

Table S1. Additional experimental information such as growth media, supplements, antibodies and primers used in this study

Table S3. Cell-wise meta information of scRNA-seq dataset, related to Figure 2

Table S4. Marker gene expression based on the Louvain cluster in scRNA-seq data

Table S5. Results of time-association analysis and average expression of significantly associated genes, related to Figure 2

Table S6. Result of the clustering and average gene expression from day 0–6 (DE), and day 6 to day 10 (FE), related to Figure 3

Table S7. Average expression of cells per bin ranked by adjusted p-value from the diffxpy analysis across the lung and hepatocyte trajectory, related to Figure 4

Table S8. Gene expression correlations to EOMES expression levels for days 0 to day 6, related to Figure S3
