# Supplementary material for: Human pluripotent stem cell fate trajectories toward lung and hepatocyte progenitors
Source: iScience. 2023 Oct 14;26(11):108205. doi: 10.1016/j.isci.2023.108205 (PMC10663741; doi:10.1016/j.isci.2023.108205)
Supplement: Document S1. Figures S1–S5 and Table S2 [file mmc1.pdf]

iScience, Volume *26*

Supplemental information

Human pluripotent stem cell fate trajectories toward lung and hepatocyte progenitors

Chaido Ori, Meshal Ansari, Ilias Angelidis, Ruth Olmer, Ulrich Martin, Fabian J. Theis, Herbert B. Schiller, and Micha Drukker

SUPPLEMENTARY FIGURES

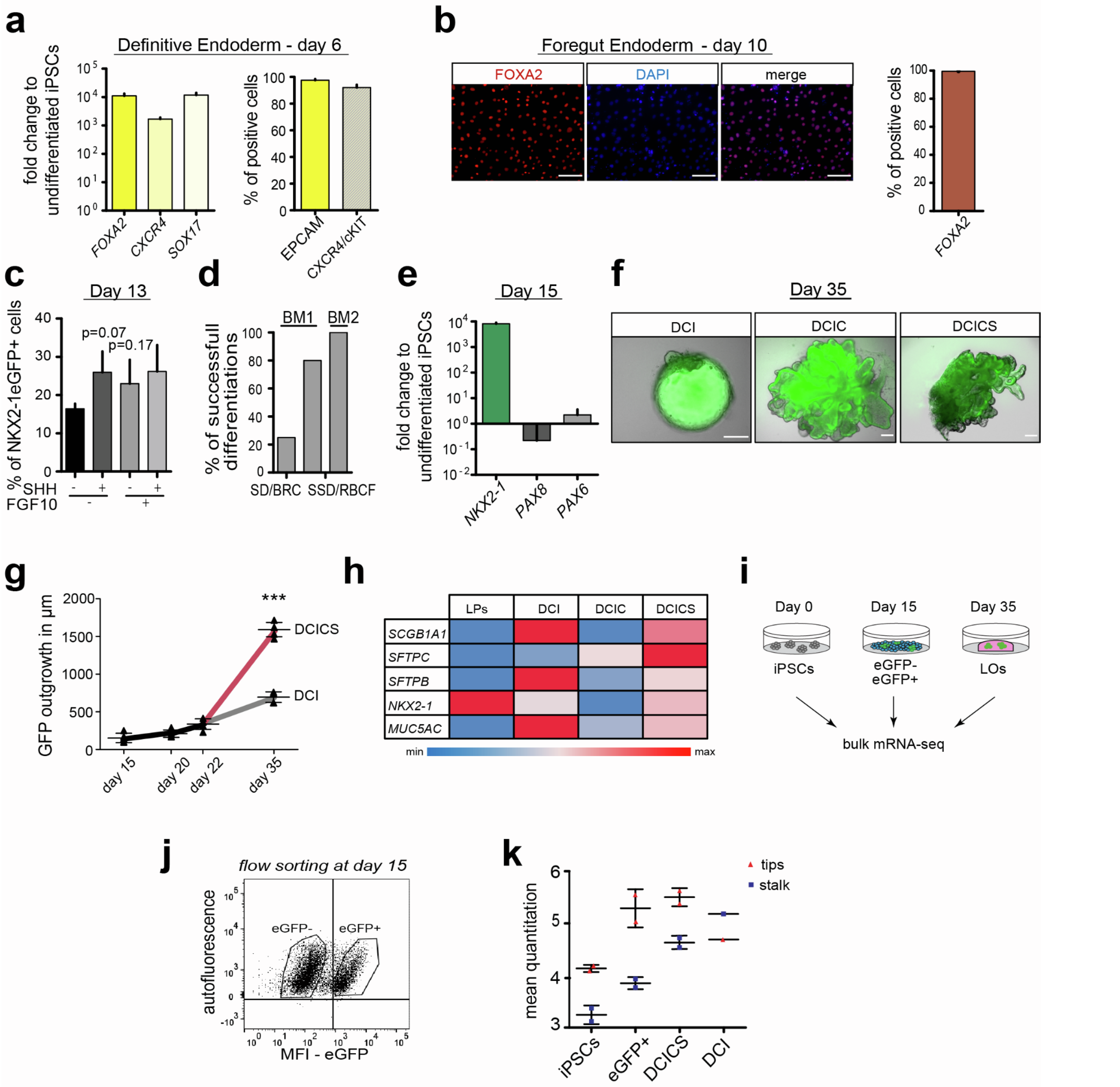

Figure S1. Differentiation of lung progenitors from human induced pluripotent stem cells, related to Fig. 1. (a) The differentiation of DE precursor cells analyzed by RT-qPCR $2^{(-\Delta\Delta CT)}$ and flow cytometry of cell surface markers on day 6 of differentiation (undifferentiated cells were used for normalization; error bars represent mean +SD, n=3). **(b)** Representative microscopic images and quantification of foregut precursors analyzed by immunostaining of FOXA2 and DNA staining (DAPI in blue) on day 10 of differentiation (error bars represent mean +SD, scale bars: 100 µm). **(c)** Quantification of NKX2-1-eGFP+ cells analyzed on day 13 of differentiation by flow cytometry; treatment by SHH and FGF10 as indicated (bars represent mean + SD, n=3 biological replicates). **(d)** Percentage of successful differentiation experiments with the indicated conditions corresponding to **Fig. 1b**. **(e)** Analysis of markers representing lung, thyroid and forebrain tissues *NKX2-1*, *PAX8* and *PAX6* respectively by RT-qPCR $2^{(-\Delta\Delta CT)}$ (error bars represent mean +SD; n=4 biological replicates). **(f-h)** Fluorescent microscopy images **(f)** and organoid growth curves **(g)**, and RT-qPCR, **(h)** of markers characteristic of the early lung (color-scaled per row). Organoids were created from day 15 colonies using conditions outlined in **Fig. 1a** of NKX2-1-eGFP+ cells, and were analyzed on day 35 (scale bars: 200 µm in **f**; RT-qPCR $2^{(-\Delta CT)}$; error bars represent mean ±SD, n=5 biological replicates; ***p ≤ 0.001 unpaired, one-tailed t-test). **(i)** Schematic illustration of samples prepared for bulk mRNA-sequencing, and **(j)** representative flow cytometry plots with the gating strategy used for sorting eGFP+ and eGFP- cells (undifferentiated cells were used as negative controls to set gates). **(k)** A plot displaying the correlation of the mean quantitation for each data store to tip (red) and stalk (blue) gene signatures from Nikolić et al.[1]

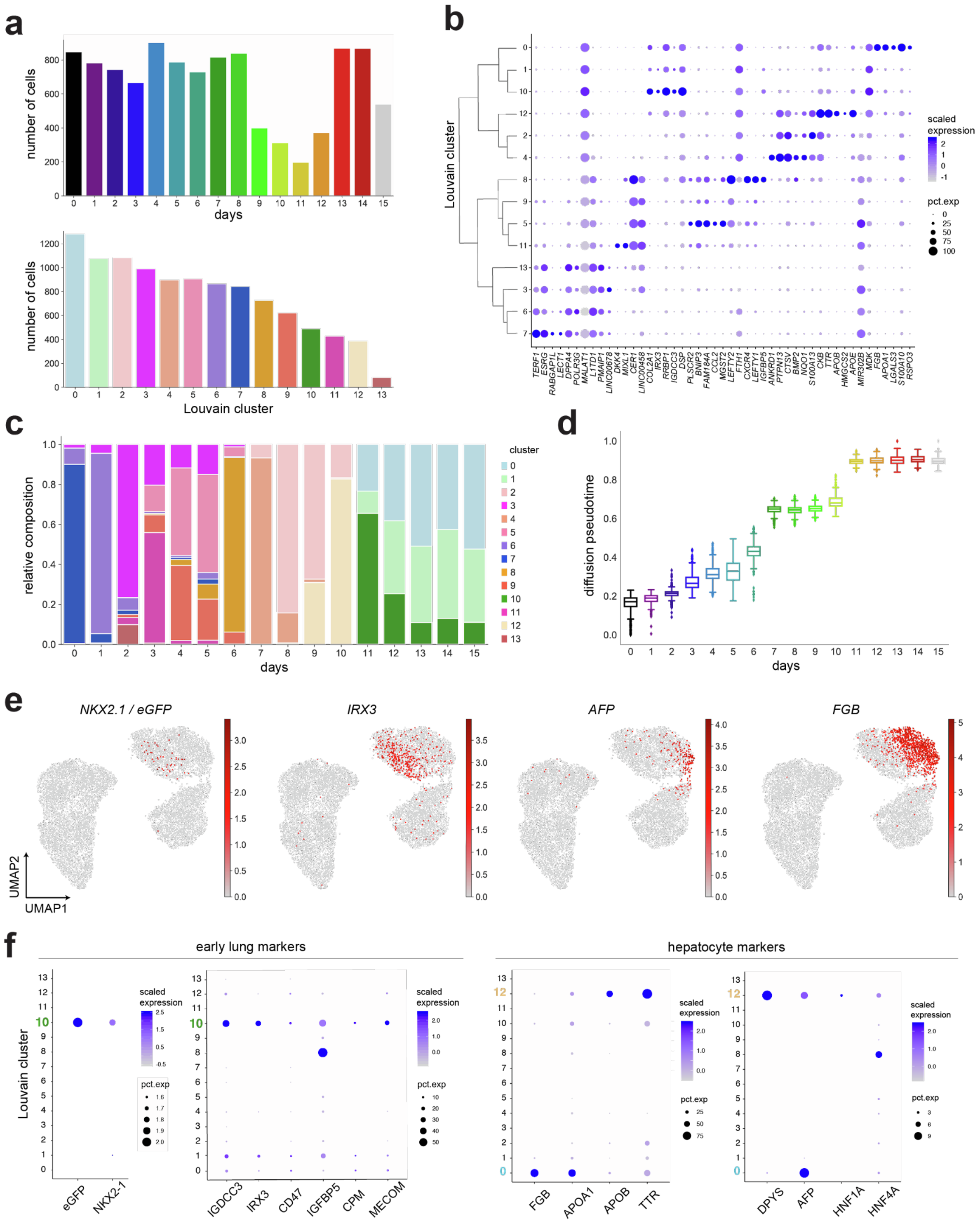

Figure S2. Overview of the single cell dataset after filtering, related to Fig. 2. (a) Barplots displaying the number of cells included in downstream analysis after quality control filtering (details in the experimental procedures) per time point (top), and Louvain cluster (bottom). **(b)** Top 5 genes per cluster shown in a dendrogram created by unsupervised clustering displaying the normalized expression level (across clusters in blue) and the percentage of cells expressing the respective gene according to dot size. **(c)** A barplot showing the Louvain cluster composition according to time point (day) of sampling. **(d)** A boxplot of diffusion in the pseudotime of all sampling time points with boxes representing the interquartile range, horizontal line the median, and the whiskers representing 1.5 times the interquartile range **(e)** UMAPs overlaid with cells expressing lung progenitor, *NKX2-1* and *IRX3*, and liver markers AFP and *FGB*, showing the separation of the two lineages. **(f)** A dot plot indicating that markers of the early lung show higher expression levels in Louvain clusters 10 and 1 than markers of hepatocytes 0 and 12.

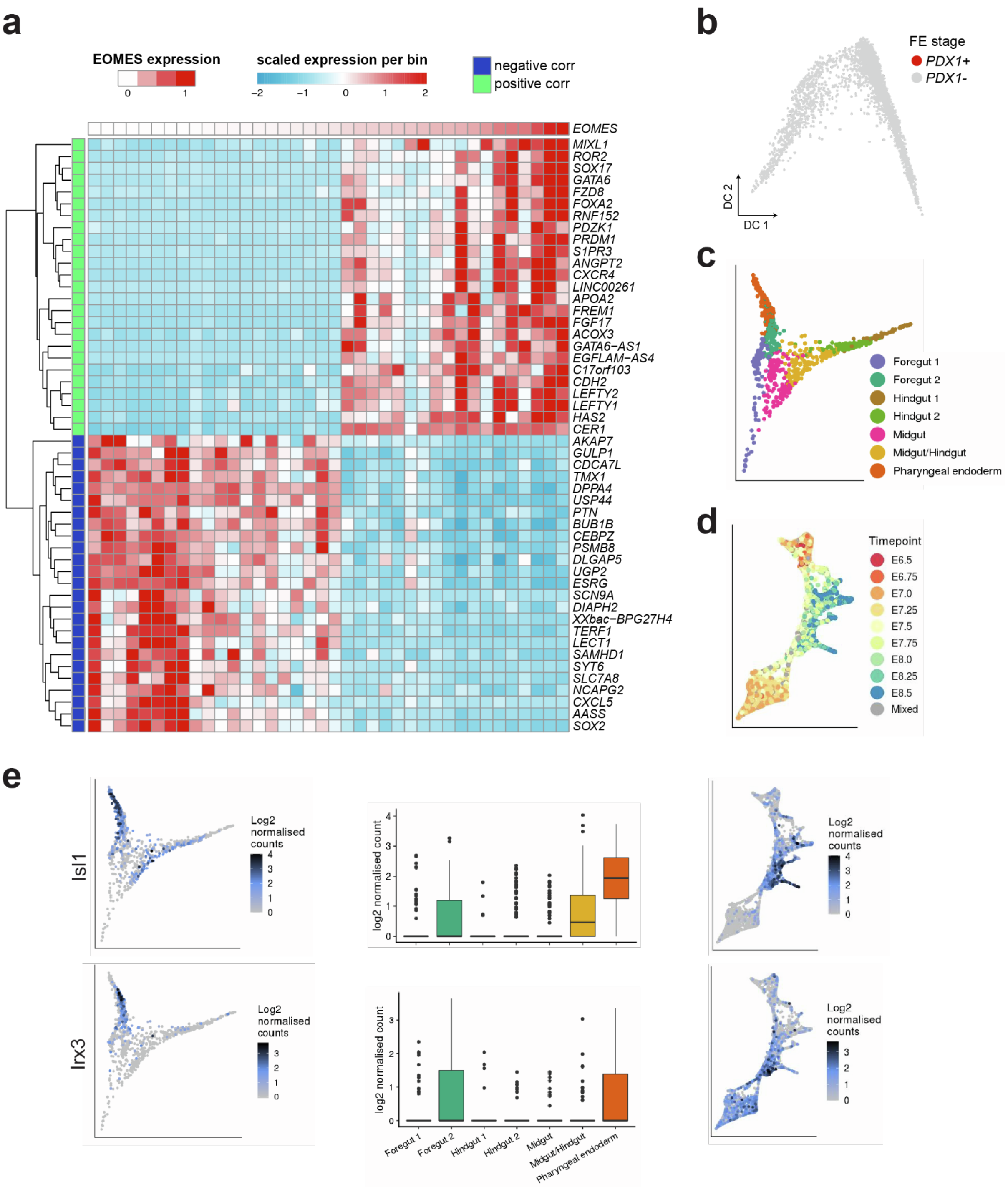

Figure S3. Correspondence to scRNA-seq data of mouse embryo during embryogenesis, related to Fig. 3. (a) A heatmap encompassing top 25 correlated and anti-correlated genes to *EOMES* expression levels for the respective days 0 to day 6. Cells were clustered into 38 bins based on transcriptomic profile, scaled average expression per bin is shown ordered according to *EOMES* expression. This revealed an association of *EOMES* with *LEFTY1*, *LEFTY2*, *CER1*, and *ROR2*, indicating active EOMES-Activin/Nodal circuit.[2] **(b)** Diffusion map of single cells for days 6 to day 10 as in Fig. 3f, showing that there were no cells positive for PDX1 at this stage. **(c-e)** Analysis that is based on the scRNA seq database of the mouse embryo during organogenesis,[3] including **(d)** representative time points of embryo collection and **(e)** the gene expression levels and tissue association of *Isl1* and *Irx3*.

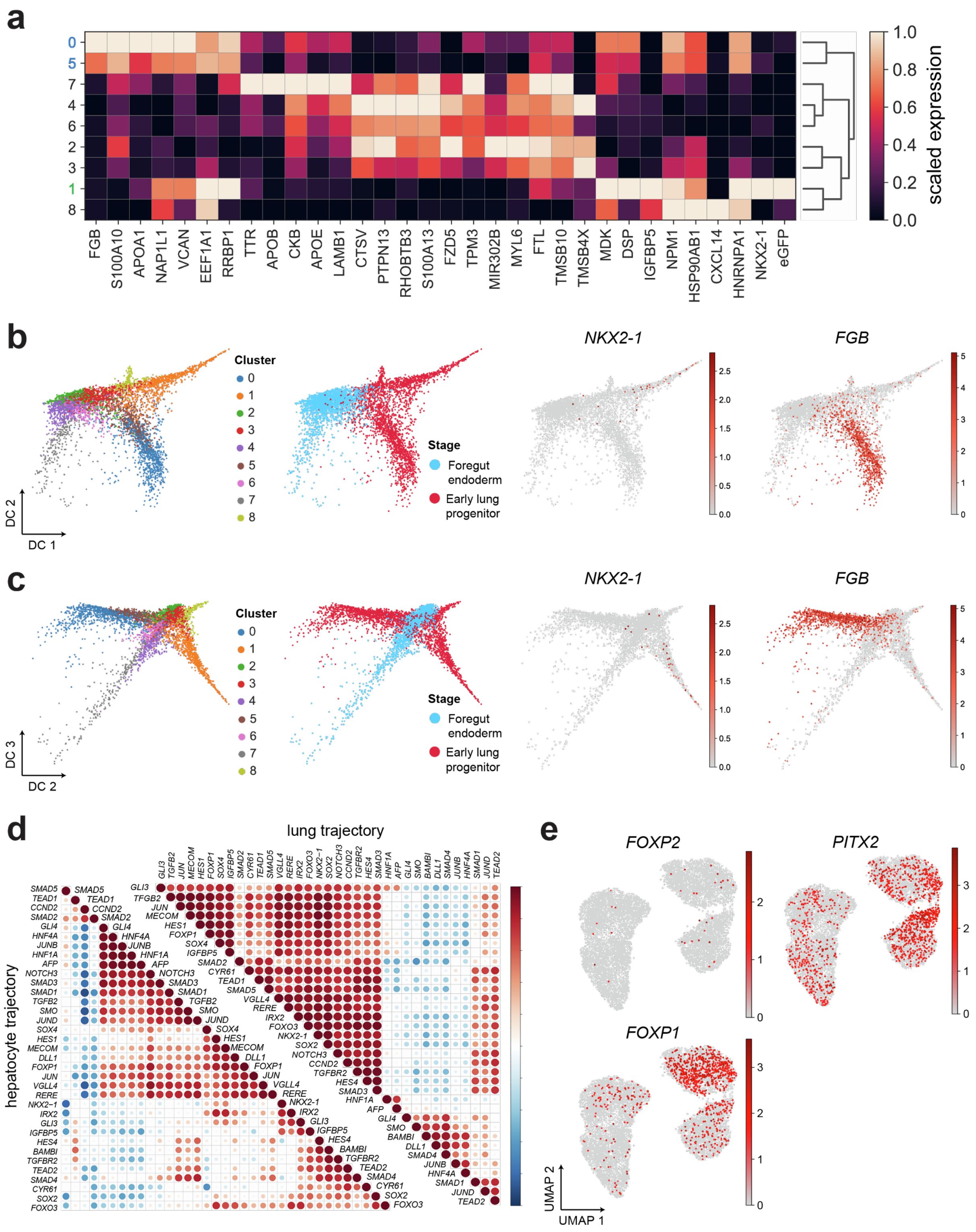

Figure S4. Diffusion map of all cells from days 7-15 of differentiation reveals minor branches that were not included in Fig 4. (a) Unsupervised clustering of the top 5% DE genes in 9 Louvain clusters. **(b,c)** Diffusion maps showing that the lung marker *NKX2-1* (and *NKX2-1-eGFP*) was restricted to sub branch #1, and hepatocyte marker *FGB* was enriched in the branches encompassing clusters 0 and 5. Cluster colors in **(a)** correspond to lung (green) and liver (blue) branches in **Fig. 4**. Diffusion map with diffusion components 1 vs. 2, and 2 vs. 3 are shown in **(b)** and **(c)** respectively of cells from day 7 to 15 coloured by louvain cluster (left) and stage (middle-left). **(d)** Pairwise gene pearson correlation profiles along the lung and liver pseudotime trajectories. **(e)** UMAP of all time points indicating expression levels of *FOXP2*, *PITX2* and *FOXP1*.

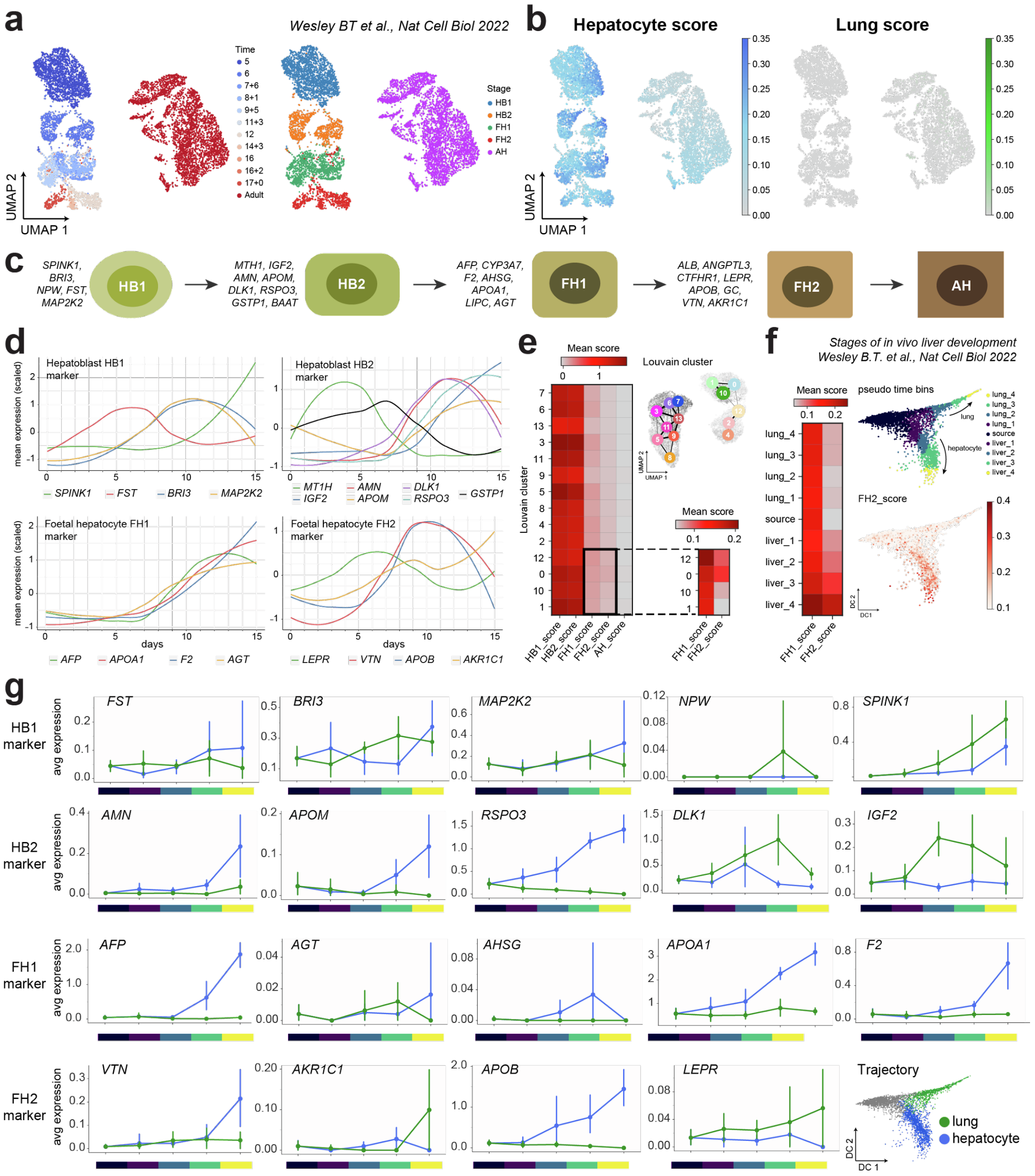

Figure S5. Comparison of iPSC-hepatoblast differentiation trajectory with scRNA-seq data of primary human liver development identifies hepatocyte-specific features, related to Fig 4. (a) UMAP embedding of the human primary liver scRNA-seq data. Hepatocyte developmental trajectory colored by the post-conception week time point and stage development (annotation as published in Wesley et al. [4] Primary liver developmental stages were categorized into hepatoblast stages 1 and 2 (HB1, HB2), fetal hepatocyte stages 1 and 2 (FH1, FH2) and adult hepatocytes (AH). **(b)** UMAP embedding of liver development scRNA-seq data overlaid with hepatocyte and lung scores per definition in Fig 4c, d. **(c)** Scheme of hepatocyte differentiation stages and characteristic genes as suggested by Wesley et al. and **(d)** the mean expression of developmental liver marker genes in our study over the course of 16 days. **(e)** Average score of the top 100 stage-specific liver marker genes grouped by louvain cluster in Fig. 2. **(f)** Matrixplot showing average score of the two fetal hepatocyte stages in our FE and ELP branching subset and corresponding to the diffusion map. **(g)** Line plots of the proposed hepatocyte stage-specific genes expression in the respective branches of our data set, ordered by the binned pseudotime as shown in Fig 4f. Line plot is shown if the respective gene is expressed in 50+ cells.

SUPPLEMENTARY TABLES

Table 2. Quality metrics of time series scRNA-seq data, related to Fig. 2.

sample_id	time_point	n_counts (median)	n_genes (median)	percent_mito (mean)	# cells
muc6865	day 0	1205.5	772.0	0.088	847
muc6866	day 1	1365.0	855.5	0.078	782
muc6867	day 2	1572.0	871.5	0.095	743
muc6868	day 3	1532.0	822.0	0.079	666
muc6869	day 4	951.0	626.0	0.082	902
muc6870	day 5	1059.0	654.5	0.068	787
muc6871	day 6	831.0	574.0	0.080	729
muc6872	day 7	1770.0	1083.5	0.057	816
muc6873	day 8	1070.0	747.5	0.064	839
muc6874	day 9	517.5	395.0	0.098	399
muc6875	day 10	411.0	294.0	0.145	312
muc6876	day 11	275.0	222.0	0.053	197
muc6877	day 12	313.5	241.0	0.091	372
muc6878	day 13	899.5	608.0	0.062	869
muc6879	day 14	876.5	590.5	0.054	868
muc6880	day 15	664.0	449.5	0.080	539

SUPPLEMENTAL REFERENCES

1. Nikolić, M. Z. *et al*. Human embryonic lung epithelial tips are multipotent progenitors that can be expanded in vitro as long-term self-renewing organoids. *Elife* **6**, (2017).

2. Teo, A. K. K. et al. Pluripotency factors regulate definitive endoderm specification through eomesodermin. Genes Dev. 25, 238–250 (2011).

3. Pijuan-Sala, B. *et al*. A single-cell molecular map of mouse gastrulation and early organogenesis. *Nature* **566**, 490–495 (2019).

4. Wesley, B. T. *et al*. Single-cell atlas of human liver development reveals pathways directing hepatic cell fates. *Nat. Cell Biol*. **24**, 1487–1498 (2022).
